# Effect of Combination l-Citrulline and Metformin Treatment on Motor Function in Patients With Duchenne Muscular Dystrophy: A Randomized Clinical Trial

**DOI:** 10.1001/jamanetworkopen.2019.14171

**Published:** 2019-10-30

**Authors:** Patricia Hafner, Ulrike Bonati, Andrea Klein, Daniela Rubino, Vanya Gocheva, Simone Schmidt, Jonas Schroeder, Günther Bernert, Vincent Laugel, Maja Steinlin, Andrea Capone, Monika Gloor, Oliver Bieri, Lars G. Hemkens, Benjamin Speich, Thomas Zumbrunn, Nuri Gueven, Dirk Fischer

**Affiliations:** 1 Division of Pediatric Neurology, University Children's Hospital Basel, Basel, Switzerland; 2 Division of Neurology, Medical University Clinic, Kantonsspital Baselland, Bruderholz, Switzerland; 3 Division of Pediatric Neurology, University of Berne Hospital, Berne, Switzerland; 4 Division of Pediatric Neurology, Lausanne University Hospital, Lausanne, Switzerland; 5 Department of Neurology, University Hospital Basel, Basel, Switzerland; 6 Department of Pediatrics, Kaiser Franz Josef Hospital, Vienna, Austria; 7 Department of Pediatric Neurology, Strasbourg University Hospital, Strasbourg, France; 8 Division of Pediatric Neurology, Children's Hospital, Aarau, Switzerland; 9 Division of Radiological Physics, Department of Radiology, University Hospital Basel, Basel, Switzerland; 10 Basel Institute for Clinical Epidemiology and Biostatistics, Department of Clinical Research, University Hospital Basel, Basel, Switzerland; 11 Clinical Trial Unit, Department of Clinical Research, University Hospital Basel, Basel, Switzerland; 12 Pharmacy, School of Medicine, University of Tasmania, Hobart, Tasmania, Australia

## Abstract

**Importance:**

Nitric oxide precursors, such as the amino acid l-arginine and the biguanide antidiabetic drug metformin, have been associated with metabolism and muscle function in patients with Duchenne muscular dystrophy (DMD). The treatment of DMD remains an unmet medical need.

**Objective:**

To evaluate the benefits and harms of a combination of l-citrulline and metformin treatment among patients with DMD.

**Design, Setting, and Participants:**

A single-center randomized double-blind placebo-controlled parallel-group clinical trial was conducted between December 12, 2013, and March 30, 2016, at the University Children’s Hospital Basel in Switzerland. A total of 47 ambulant male patients aged 6.5 to 10 years with genetically confirmed DMD were recruited locally and from the patient registries of Switzerland, Germany, Austria, and France. Data were analyzed from April 6, 2016, to September 5, 2019.

**Interventions:**

Patients in the treatment group received 2500 mg of l-citrulline and 250 mg of metformin (combination therapy) 3 times a day for 26 weeks compared with patients in the control group, who received placebo.

**Main Outcomes and Measures:**

The primary end point was the change in transfer and standing posture, as assessed by the first dimension of the Motor Function Measure, version 32, from baseline to week 26. Secondary end points included assessments of timed function, quantitative muscle force, biomarkers for muscle necrosis, and adverse events. The 2 prespecified subgroups comprised patients who were able to walk 350 m or more in 6 minutes (stable subgroup) and patients who were not able to walk 350 m in 6 minutes (unstable subgroup) at baseline.

**Results:**

Among 49 ambulant male children with DMD who were screened for eligibility, 47 patients with a mean (SD) age of 8.2 (1.1) years were randomized to a treatment group receiving combination therapy (n = 23) or a control group receiving placebo (n = 24), and 45 patients completed the study. No significant differences between groups were found in the results of timed function and muscle force tests for overall, proximal and axial, and distal motor function. Among patients receiving combination therapy, the Motor Function Measure first dimension subscore decrease was 5.5% greater than that of patients receiving placebo (95% CI, −1.0% to 12.1%; *P* = .09). The administration of combination therapy had significantly favorable effects on the first dimension subscore decrease among the 29 patients in the stable subgroup (6.7%; 95% CI, 0.9%-12.6%; *P* = .03) but not among the 15 patients in the unstable subgroup (3.9%; 95% CI, −13.2% to 20.9%; *P* = .63). Overall, the treatment was well tolerated with only mild adverse effects.

**Conclusions and Relevance:**

Treatment with combination therapy was not associated with an overall reduction in motor function decline among ambulant patients with DMD; however, a reduction in motor function decline was observed among the stable subgroup of patients treated with combination therapy. The statistically nonsignificant difference of distal motor function in favor of combination therapy and the reduced degeneration of muscle tissue appear to support the treatment concept, but the study may have lacked sufficient statistical power. Further research exploring this treatment option with a greater number of patients is warranted.

**Trial Registration:**

ClinicalTrials.gov identifier: NCT01995032

## Key Points

**Question:**

Does treatment with l-citrulline and metformin combination therapy reduce motor function decline in ambulant patients with Duchenne muscular dystrophy?

**Findings:**

In this randomized clinical trial of 47 ambulant male children aged 6.5 to 10 years with Duchenne muscular dystrophy, treatment with a combination of l-citrulline and metformin therapies provided a clinically relevant but not statistically significant reduction in motor function decline, as assessed by the transfer and standing posture dimension of the Motor Function Measure scale. No indications of harm were found in the intention-to-treat population.

**Meaning:**

Treatment with a combination of l-citrulline and metformin therapies may slow muscle function decline in a specific subgroup of patients with Duchenne muscular dystrophy, but additional clinical trials with greater statistical power are warranted.

## Teaser

This randomized clinical trial evaluates the effect of treatment with a combination of L-citrulline and metformin on the motor function of ambulant boys with Duchenne muscular dystrophy.

## Introduction

Duchenne muscular dystrophy (DMD) is an X-linked recessive disease that occurs in 1 of 3800 to 6000 male births.^1^ The genetic mutation results in loss of sarcolemmal dystrophin. In addition, altered neuronal nitric oxide (NO) synthase and mitochondrial dysfunction impair muscle function.^2,3,4,5^ Duchenne muscular dystrophy is characterized by the rapid and irreversible replacement of normal skeletal muscle with connective and adipose tissue, leading to a loss of motor function and muscle degeneration. Current therapeutic management remains supportive.^6^

Metformin, a well-established biguanide treatment for diabetes, improves glucose tolerance by decreasing hepatic glucose production, thereby decreasing the intestinal absorption of glucose and improving insulin sensitivity. Beneficial effects on dystrophic skeletal muscle in *mdx* mice (ie, mice with an *mdx* allele mutation that is equivalent to the mutation in the human dystrophin gene, *DMD,* which causes DMD) have been observed.^7,8^ Mantuano et al^8^ suggested that despite the observed amelioration in muscle histopathology and ex vivo diaphragm force, no clear protective actions on dystrophic muscle metabolism in *mdx* mice were observed. The lack of metabolic effects may have been owing to the inability of metformin treatment alone to increase the low muscle levels of the amino acids l-arginine and l-citrulline and the amino sulfonic acid taurine, supporting the therapeutic combination of metformin with NO sources. The amino acid l-citrulline is largely converted to l-arginine in the kidneys.^9^ The intake of l-citrulline in humans leads to higher peak l-arginine and NO concentrations compared with equivalent l-arginine doses and the intake of l-arginine itself, and single oral doses of up to 15 g of l-citrulline have been well tolerated by patients without adverse effects (AEs).^10^ In addition, l-citrulline reduces muscle necrosis in patients with low protein intake.^11,12^ It has been suggested that l-citrulline has protective effects on muscle protein metabolism, mediated through NO synthase.^13^ The significantly reduced muscle content of NO precursors found in *mdx* mice, combined with the partial response of dystrophic muscle to metformin,^8^ supports the concept of a combined therapy consisting of metformin and an NO precursor, such as l-citrulline, to modify NO levels and mitochondrial metabolism and thereby ameliorate muscle function in patients with DMD. To our knowledge, no other clinical trial has evaluated the treatment of patients with DMD using a combination of l-citrulline and metformin therapies. We aimed to evaluate the benefits and harms of treatment with combination therapy among children with DMD in a randomized clinical trial.

## Methods

### Study Design and Participants

**Figure 1.**
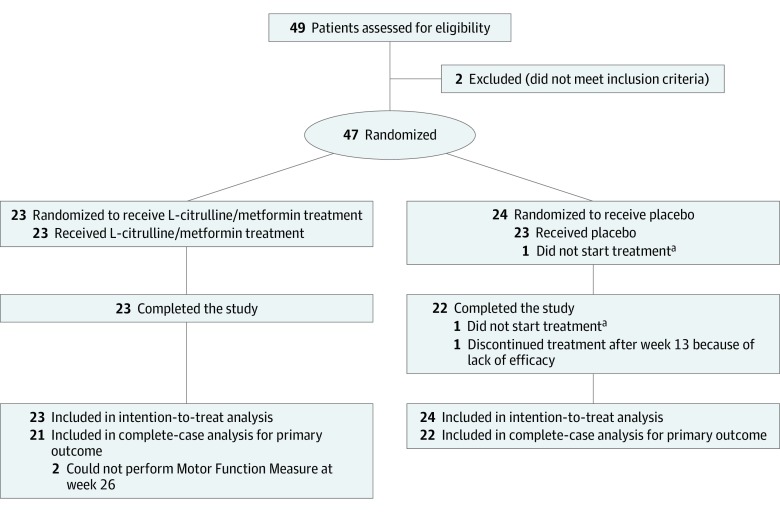
CONSORT Flow Diagram ^a^Indicates the same patient.

We performed a single-center randomized (1:1 ratio) double-blind placebo-controlled parallel-group study in an outpatient setting (Figure 1). Patients were enrolled from December 12, 2013, to September 22, 2015, and were recruited during routine care visits at the pediatric outpatient clinic of the University Children’s Hospital Basel in Switzerland, which is a secondary referral center for pediatric neuromuscular diseases. We also screened the DMD patient registries of Switzerland, Germany, Austria, and France for eligible patients. The clinical trial was conducted in accordance with the Declaration of Helsinki^14^ and the Guideline for Good Clinical Practice,^15^ and it was approved by the local ethics committee (the ethics committee of both Basel cantons) and the Swiss Agency for Therapeutic Products (Swissmedic). We followed the guidelines of the European Medicines Agency^16,17^ for the conduct and design of the study. The clinical trial was monitored by independent organizations (the Clinical Trial Unit of University Hospital Basel and Kammermann Monitoring Services GmBh Zug) and followed the Consolidated Standards of Reporting Trials (CONSORT) guidelines. The study protocol has been published previously,^16,18^ and the original protocol is available in Supplement 1. All examinations were performed at the University Children’s Hospital Basel. Patients and parents were informed about preclinical evidence, alternative treatments, and possible benefits and harms associated with the study at the screening visit. Oral informed assent from all children and written informed consent from all parents were obtained. Data were analyzed from April 6, 2016, to September 5, 2019.

We included patients aged 6.5 to 10 years with genetically confirmed DMD who were able to walk at least 150 m in 6 minutes and had moderate motor function (≥40%, as measured by the first dimension [D1; pertaining to transfer and standing posture] of the Motor Function Measure [MFM] scale). Patients who received the combination therapy or their l-arginine metabolites within the last 3 months were excluded from participation to eliminate interfering effects. Also excluded were patients who (1) had received an unstable corticosteroid treatment within the last 6 months; (2) were participants in other clinical trials; (3) had known intolerance or hypersensitivity to any study treatment; (4) had other chronic diseases or clinically relevant limitations of renal, liver, or heart function; or (5) had a diagnosis of cancer or suspected cancer. No additional eligibility criteria were applied.

### Interventions and Outcomes

Eligible patients were randomized in a 1:1 ratio to either the combination therapy group or the placebo control group by an independent researcher using a computer-generated randomization list. Children in the treatment group received 2500 mg of l-citrulline (via powder in sachets dissolved in water) and 250 mg of metformin (via metformin hydrochloride tablets). Children in the control group received placebo consisting of mannitol administered via matching powder and tablets. Treatment was administered 3 times daily over 26 weeks. Returned medication was counted at follow-up visits. No change in concomitant care or usual practice occurred during the study period.

The primary outcome was the change in patients’ ambulation after 26 weeks of treatment, as assessed by the D1 subscore (range, 0-39, with 0 indicating no standing and transfer function and 39 indicating normal motor function; the result is expressed as a percentage of the maximum possible score) on the MFM-32 scale, which consists of 32 items that evaluate patients’ motor function in lying, sitting, and standing positions; the MFM tool has been validated for use in children.^19^ The D1 items are associated with the loss of ambulation in children with DMD, and the scale effectively represents short-term (ie, 3-month) changes in motor function.^20^

Secondary outcomes of the study were changes in the following parameters: (1) overall motor function (measured by the total MFM score); (2) proximal and axial motor functions (measured by the MFM D2 [second dimension] subscore); (3) distal motor function (measured by the MFM D3 [third dimension] subscore); (4) motor function related to the speed of movement (measured by timed function tests); (5) 6-minute walking distance (6-MWD);^21,22^ (6) knee extension and elbow flexion (assessed by handheld dynamometry and quantitative muscle testing) as measures of muscle force independent of muscle function and aspect; and (7) biomarkers of muscle necrosis in plasma and urine. Biomarkers in plasma included creatine kinase and creatine-kinase-MB isoform, aspartate aminotransferase, alanine aminotransferase, l-lactate dehydrogenase, gamma-glutamyl transferase, bilirubin, alkaline phosphatase, and creatinine; biomarkers in urine included summated branch chain amino acids, summated essential and nonessential amino acids, and changes in safety-related laboratory markers (complete blood cell count, renal and liver function tests, and sodium, potassium, chloride, calcium, and phosphate levels). The last 7 thigh muscle tissue changes detected through magnetic resonance imaging (MRI), using fat fraction (FF) and transverse relaxation time (T2)–weighted images, were evaluated.^20,23,24^ All MRI examinations were performed using a 3-T MRI scanner (Siemens Healthcare) consisting of two 16-element body array coils and 1 spine coil. One independent MRI technologist, who had more than 15 years of experience in the use of MRI evaluations for research purposes, performed all examinations according to a prespecified protocol.

Localization assessments comprised a series of scout images taken in 3 orthogonal directions and additional scout images taken parallel and orthogonal to the femur as well as through the hip and knee joint space. A single axial volume halfway between the knee and hip joints was selected for image acquisition, from which multiple sections were reconstructed. For each patient, regions of interest were manually drawn over the thigh muscles depicted on the axial images, and care was given to areas with chemical shift artifacts. The 3 regions of interest chosen for evaluation were the knee extensors (quadriceps), knee flexors (hamstrings), and adductor muscle groups in both legs. The MRI analyses at baseline and week 26 were performed blinded and after completion of the clinical trial. The 2-point Dixon method was used, in which 2 images at identical positions were acquired so that water and fat protons could be observed through in-phase and opposed-phase imaging, respectively. A combination of these images yielded combined water and fat images. Relative fat content maps were generated from the pixelwise FF, which was calculated by dividing the fat content by the fat plus water content. The regions of interest were placed on the maps, and the mean fat content was calculated for each muscle group. With the exception of MRI evaluations, which were performed only at baseline and week 26, all other outcomes were measured at 13 and 26 weeks after randomization.

### Sample Size, Randomization, and Masking

The sample size estimation was based on findings from a pilot study of 5 patients with DMD^18^ and data from a study by Vuillerot et al,^25^ which reported that the natural progression of DMD in children older than 6 years was associated with an annual change in the MFM D1 subscore of −17.2%. Data indicated an early acceleration of motor function followed by a deceleration of motor decline and a loss of ambulation. To simplify, we assumed a consistent decline owing to the natural progression of the disease.^26^ At least 21 patients per group were needed to detect whether treatment with combination therapy reduced this motor function decline by at least 50% over 26 weeks (ie, a −4.3% change in ambulation in the treatment group vs a −8.6% change in the control group, as measured by the MFM D1 scale), with a 2-sided significance of α = .05 and a power of 0.8. A dropout rate of 10% was assumed, and a total of 47 patients were enrolled.^16^

Patients were randomly allocated to the treatment and control groups using an initial unbalanced group of 5 patients and subsequent randomly permuted balanced groups of 2 or 4 patients.^27^ An independent pharmacist dispensed either active or placebo compounds using a computer-generated randomization list. The l-citrulline powder and matching placebo powder were prepared in identical sachets and prepackaged in cardboard boxes; the metformin tablets and matching placebo tablets were dispensed in identical bottles. The cardboard boxes and bottles were consecutively numbered for each participant based on the randomization schedule. All study personnel, including study coordinators, study nurses, physiotherapists, and investigators, as well as participants, caregivers, and outcome assessors, were blinded to treatment allocation.

### Statistical Analysis

The primary analyses were based on the intention-to-treat (ITT) principle; thus, the effects of treatment with combination therapy were compared with placebo in all randomized patients for whom a baseline measurement of the primary end point was available (the ITT population). The primary and secondary end points were modeled through an analysis of covariance, using the measurement at week 13 or week 26 as the dependent variable, the measurement at baseline as the covariate, and the group allocation (treatment or control) as the independent variable. To impute missing outcome values, we performed multiple imputations (n = 9999) through chained equations using predictive mean matching that incorporated all variables of the linear models underlying the analysis of covariance.

Two subgroup analyses were conducted. The first aimed to explore potentially different treatment effects among patients who were at different stages of the disease. The natural course of motor development and decline in patients with DMD comprises an initial improvement in function, then a stable plateau followed by a phase of consistent motor function decline. We evaluated patients based on their ability to walk 350 m or more (stable subgroup) or less than 350 m (unstable subgroup) in 6 minutes. The homogeneity of patients in the stable subgroup differed from that of patients in the unstable subgroup.^28,29,30,31^ The rate of motor decline in patients able to walk 350 m or more was relatively constant, while the motor function of patients unable to walk 350 m either continued to improve (if they were very young and still gaining motor function) or soon declined until they lost the ability to ambulate freely. The same procedure used to analyze the primary and secondary end points was used to analyze each subgroup. This subgroup analysis was prespecified in the statistical analysis plan before the end of the clinical trial and the unblinding of the data.

An additional subgroup analysis was performed post hoc to evaluate patients who did and did not receive corticosteroid treatment before randomization. This subgroup analysis was not prespecified in the statistical analysis plan. Corticosteroid therapy is considered a standard treatment in this patient population and has been associated with better outcomes; however, this treatment is often refused by parents and patients owing to concerns about AEs. In this subgroup analysis, we explored potential treatment interactions and addressed confounding variables that may have been present owing to the unbalanced proportions of patients who received corticosteroids before study treatment despite randomization.

To assess the robustness of the results, sensitivity analyses were conducted for all of the outcomes in the safety population (a subset of the ITT population that comprised all patients who were randomized and received ≥1 dose of combination therapy) and the complete-case population (a subset of the safety population that comprised all patients who were included in the complete follow-up analysis for the primary end point; eTable 1 in Supplement 2). In addition, we performed sensitivity analyses using other methods, such as complete-case and last-observation-carried-forward techniques, to address missing data. All computations were performed with R, version 3.6.1 (R Foundation for Statistical Analysis). All statistical tests were 2-sided with a significance level of α = .05.

## Results

A total of 49 ambulant male patients aged 6.5 to 10 years with DMD were screened between December 12, 2013, and March 30, 2016. Of those, 47 patients with a mean (SD) age of 8.2 (1.1) years were randomized to the combination therapy group (n = 23) or the placebo control group (n = 24); 45 patients completed the study, with no missing data. Of the 2 patients who did not complete the study, 1 did not reach an MFM D1 subscore of 40%, and the other was unable to complete the MFM evaluation or perform the 6-MWD. Baseline characteristics were comparable in the treatment and control groups, with the exception of corticosteroid use (Table 1). In both groups, 2 patients had begun corticosteroid treatment between 6 and 12 months before randomization. Two patients in the control group withdrew consent, 1 before initiating study treatment and 1 owing to a lack of treatment efficacy after 13 weeks. Two patients in the treatment group sustained a bone fracture; 1 patient fractured his femur on the right side, which made it impossible to assess his motor function at weeks 13 and 26, and the other patient fractured his tibia, which resulted in the inability to perform the MFM D1 evaluation at week 26. The 45 remaining patients completed 26 weeks of follow-up to assess the primary outcome and were included in the complete-case analysis, and all of the initial 47 patients were included in the ITT analysis (Figure 1).

**Table 1.** Demographic and Baseline Characteristics of Intention-to-Treat Population

Characteristic	Mean (SD)	
	Combination Therapy Group (n = 23)	Placebo Group (n = 24)
Age, y	8.2 (1.2)	8.2 (1.0)
Weight, kg	27.5 (7.1)	27.4 (6.2)
Height, m	1.3 (0.1)	1.3 (0.1)
BMI	17.6 (3.4)	17.3 (2.7)
Corticosteroid treatment, No. (%)	12 (52.2)	21 (87.5)
Plasma, μmol/L		
l-Citrulline^a^	19.6 (5.2)	19.1 (4.6)
l-Arginine^a^	66.1 (24.0)	66.6 (17.1)
l-Ornithine^a^	80.4 (20.1)	73.4 (16.6)
MFM score, %		
Total	79.5 (7.4)	78.8 (5.6)
D1	60.1 (11.9)	58.0 (10.9)
D2	95.7 (5.3)	95.6 (4.0)
D3	89.9 (9.0)	89.7 (8.6)
6-MWD, m	362.0 (94.7)	356.7 (55.3)
Supine uptime, s	10.1 (6.6)	10.4 (6.3)
10-m walk test, s	6.7 (2.2)	6.8 (1.5)
Strength, N	164.8 (71.6)	156.4 (51.8)
MRI of muscle groups^b^		
FF, %	27.7 (12.0)	26.3 (13.3)
T2, ms	47.5 (6.5)	46.5 (6.7)

Abbreviations: BMI, body mass index (calculated as weight in kilograms divided by height in meters squared); combination therapy, l-citrulline and metformin treatment; D1, first dimension of the MFM, referring to transfer and standing posture; D2, second dimension of the MFM, referring to proximal and axial motor functions; D3, third dimension of the MFM, referring to distal motor function; FF, fat fraction; MFM, Motor Function Measure; MRI, magnetic resonance imaging; 6-MWD, 6-minute walking distance; T2, transverse weighted relaxation time.

^a^ Reference ranges for l-citrulline are 18 to 50 μmol/L; for l-arginine, 38 to 98 μmol/L; and for l-ornithine, 24 to 64 μmol/L.

^b^ The muscle groups evaluated were knee extensors, knee flexors, and abductors in both legs.

**Figure 2.**
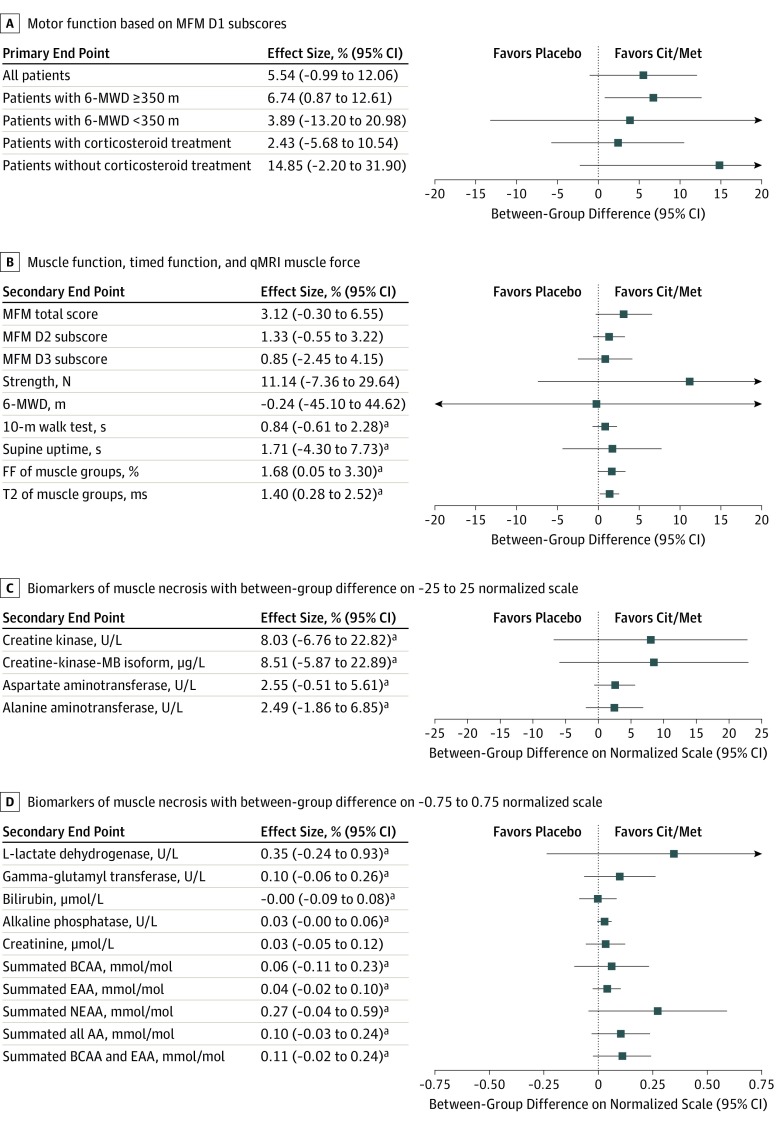
Effect Sizes for Primary End Point and Selected Secondary End Points In end points for which a decrease indicated an improvement of a test result, a sign reversal was performed to allow comparison. AA indicates amino acids; BCAA, branch chain amino acids; Cit/Met, citrulline and metformin combination therapy; D1, first dimension of the MFM, referring to transfer and standing posture; EAA, essential amino acids; FF, fat fraction; MFM, Motor Function Measure; NEAA, nonessential amino acids; qMRI, quantitative magnetic resonance imaging; 6-MWD, 6-minute walking distance; and T2, transverse weighted relaxation time.

The change of motor function, as measured by the MFM D1 scale, did not indicate a statistically significant difference at 26 weeks among patients in the treatment group (−4.0%; 95% CI, −8.7% to 0.7%) compared with those in the control group (−9.6%; 95% CI,−14.1% to −5.1%), with a mean between-group difference of 5.5% (95% CI, −1.0% to 12.1%; *P* = .09; Table 2 and Figure 2). An examination of the primary end point at week 13 (eTable 2 in Supplement 2) and week 26 (Table 2) indicates that differences between the treatment and control groups increased over time (eFigure in Supplement 2).

**Table 2.** Overall Results at Week 26 in Intention-to-Treat Population

Outcome	Combination Therapy Group (n = 23)			Placebo Group (n = 24)			Between-Group Difference	
	Mean (SD)		Change From Baseline to Week 26 (95% CI)	Mean (SD)		Change From Baseline to Week 26 (95% CI)	Difference (95% CI)	*P* Value
	Baseline	Week 26		Baseline	Week 26			
MFM score, %								
Total	79.5 (7.4)	80.1 (6.2)	0.2 (−2.2 to 2.7)	78.7 (5.5)	76.0 (7.2)	−2.9 (−5.3 to −0.5)	3.1 (−0.3 to 6.5)	0.07
D1	60.1 (11.9)	57.5 (14.0)	−4.0 (−8.7 to 0.7)	58.0 (10.8)	49.5 (14.1)	−9.6 (−14.1 to −5.1)	5.5 (−1.0 to 12.1)	0.09
D2	95.7 (5.3)	97.0 (2.3)	1.3 (−0.0 to 2.6)	95.6 (4.0)	95.6 (3.7)	−0.0 (−1.4 to 1.3)	1.3 (−0.5 to 3.2)	0.16
D3	89.9 (9.0)	93.0 (4.9)	2.8 (0.4 to 5.2)	89.7 (8.6)	91.3 (8.9)	1.9 (−0.3 to 4.2)	0.9 (−2.4 to 4.2)	0.6
Strength, N	164.8 (71.6)	171.3 (72.7)	−0.8 (−14.0 to 12.4)	156.4 (51.8)	148.9 (61.5)	−11.9 (−24.9 to 1.0)	11.1 (−7.4 to 29.6)	0.23
6-MWD, m	362.0 (94.7)	339.0 (102.3)	−30.6 (−62.4 to 1.1)	356.7 (55.3)	330.8 (89.3)	−30.3 (−62.2 to 1.6)	0.3 (−45.1 to 44.6)	0.99
10-m walk test,s	6.7 (2.2)	6.9 (2.2)	0.7 (−0.4 to 1.7)	6.8 (1.5)	7.9 (3.3)	1.5 (0.5 to 2.5)	−0.8 (−2.3 to 0.6)	0.25
Supine uptime, s	10.1 (6.6)	11.7 (8.5)	2.3 (−1.8 to 6.5)	10.4 (6.2)	12.7 (10.6)	4.1 (−0.3 to 8.4)	−1.7 (−7.7 to 4.3)	0.57
MRI of muscle groups^a^								
FF, %	27.7 (12.0)	31.7 (13.2)	3.5 (2.4 to 4.6)	26.3 (13.3)	29.7 (15.1)	5.2 (4.0 to 6.3)	−1.7 (−3.3 to −0.1)	0.04
T2, ms	47.5 (6.5)	49.5 (7.8)	1.9 (1.1 to 2.7)	46.5 (6.7)	48.6 (8.8)	3.3 (2.5 to 4.1)	−1.4 (−2.5 to −0.3)	0.02
6-MWD at baseline^b^								
≥350 m	67.1 (9.2)	63.7 (9.8)	−2.74 (−7.00 to 1.51)	60.7 (12.1)	52.3 (12.7)	−9.5 (−13.4 to −5.6)	6.7 (0.9 to 12.6)	0.03
<350 m	51.0 (8.6)	47.4 (14.4)	−4.86 (−16.4 to 6.8)	52.6 (4.5)	43.6 (16.1)	−8.7 (−21.4 to 4.0)	3.9 (−13.2 to 20.9)	0.63
Corticosteroid treatment^c^								
Yes	58.5 (10.8)	53.4 (13.2)	−6.3 (−12.7 to 0.2)	58.5 (11.1)	50.9 (14.3)	−8.70 (−13.6 to −3.8)	2.4 (−5.7 to 10.5)	0.55
No	61.8 (13.4)	62.1 (14.0)	−0.4 (−8.6 to 7.8)	54.7 (9.7)	41.0 (11.2)	−15.3 (−29.7 to −0.9)	14.8 (−2.3 to 32.0)	0.08

Abbreviations: Combination therapy, l-citrulline and metformin treatment; D1, first dimension of the MFM, referring to transfer and standing posture; D2, second dimension of the MFM, referring to proximal and axial motor functions; D3, third dimension of the MFM, referring to distal motor function; FF, fat fraction; MFM, Motor Function Measure; MRI, magnetic resonance imaging; 6-MWD, 6-minute walking distance; T2, transverse weighted relaxation time.

^a^ The muscle groups evaluated were knee extensors, knee flexors, and abductors in both legs.

^b^ The 6-MWD at baseline represents data from the analysis of the prespecified subgroup, which included 29 patients who were able to walk 350 m or more in 6 minutes and 18 patients who were not able to walk 350 m in 6 minutes; data are based on MFM D1 subscores (*P* for interaction = .67).

^c^ Corticosteroid treatment represents data from the analysis of the post hoc subgroup, which included 33 patients who received corticosteroid treatment before randomization and 14 patients who did not; data are based on MFM D1 subscores (*P* for interaction = .20).

No significant differences were found in the overall, proximal and axial, or distal motor function assessments, in the timed function tests, or in the muscle force evaluation after 26 weeks of treatment (Table 2).

Muscle degeneration was significantly reduced in the treatment group compared with the control group at 26 weeks, as indicated by smaller changes in the FFs and T2 weighted relaxation times from baseline, which were measured through quantitative MRI (qMRI) of the muscle groups (knee extensors, knee flexors, and abductors). The mean between-group difference in the FFs of all 3 muscle groups in both legs was −1.7% (95% CI, −3.3% to −0.1%; *P* = .04), and the mean between-group difference in the T2 weighted relaxation times of all 3 muscle groups was −1.4 milliseconds (95% CI, −2.5 to −0.3 milliseconds; *P* = .02; Table 2 and Figure 2).

There were no significant differences between the treatment group 23 patients at weeks 13 and 26 and control group 24 patients at weeks 13 and 26 in laboratory biomarkers indicative of muscle necrosis (eTable 3 and eTable 4 in Supplement 2 include outcomes at week 26 and week 13, respectively).

Prespecified subgroup analyses indicated that 13 patients in the stable subgroup who received combination therapy had a smaller, statistically significant decrease in their MFM D1 subscores after 26 weeks compared with 16 patients in the stable subgroup who received placebo (the mean between-group difference in MFM D1 subscores was 6.7%; 95% CI, 0.9%-12.6%; P = .03); there was no significant difference in MFM D1 subscores between patients in the unstable subgroup who received combination therapy vs those who received placebo (3.9%; 95%, CI −13.2% to 20.9%; *P* = .63; *P* for interaction = .67; Table 2). Post hoc subgroup analyses revealed no significant difference in the primary outcome between patients who did and patients who did not receive corticosteroid treatment before randomization (14.8%; 95% CI, −2.3% to 32.0%; *P* = .08; *P* for interaction = .20 for the group that did not receive corticosteroid treatment; Table 2).

There was no indication of relevant harm associated with combination therapy, which was well tolerated overall (Table 3). Among 17 of 23 patients (73.9%) in the treatment group and 13 of 24 patients (54.1%) in the control group, at least 1 AE was reported (relative risk, 1.4; 95% CI, 0.9-2.1). Most AEs were of mild intensity, and the most common AEs were the occurrence of mild, transient gastrointestinal symptoms, which are a known AE of metformin treatment that was observed in 4 patients (17.4%) who received combination therapy and 2 patients (8.3%) who received placebo (relative risk, 2.1; 95% CI, 0.4-10.3). Severe AEs occurred in 3 patients (13.0%) who received combination therapy and in no patients who received placebo (risk difference, 0.1; 95% CI, −0.1 to 0.3). The 3 severe AEs, which occurred exclusively in the treatment group, included 1 patient who sustained a deep cut in his leg from glass, 1 who sustained a tibia fracture, and 1 who sustained a femur fracture. Both of the bone fractures were caused by falls (1 patient had been treated with corticosteroids and the other was corticosteroid-naive). Laboratory assessments of safety did not indicate any significant changes.

**Table 3.** Adverse Events by Study Group

Adverse Event	No. (%)	
	Combination Therapy Group (n = 23)	Placebo Group (n = 24)
≥1 Event	17 (73.9)	13 (54.1)
Mild or moderate		
Gastrointestinal symptoms	4 (17.4)	2 (8.3)
Exanthema	2 (8.7)	3 (12.5)
Fall with contusion of the leg	2 (8.7)	1 (4.2)
Common cold	1 (4.3)	2 (8.3)
Vomiting	0 (0)	2 (8.3)
Swollen pharyngeal tonsils	2 (8.7)	0
Nausea	1 (4.3)	1 (4.2)
Mycosis	1 (4.3)	1 (4.2)
Flatulence	0	2 (8.3)
Distortion of the upper ankle joint	1 (4.3)	0
Fever	0	1 (4.2)
Cough	0	1 (4.2)
Inflammation of the middle ear	0	1 (4.2)
Vertigo	1 (4.3)	0
Loss of appetite	1 (4.3)	0
Epistaxis	0	1 (4.2)
Bug bite	1 (4.3)	0
Back pain	1 (4.3)	0
Severe		
Bone fracture (tibia/femur)	2 (8.7)	0
Deep cut in the leg	1 (4.3)	0
Fatal	0	0
Discontinuation of treatment		
Abnormal laboratory value	0	0
Other	0	0

All sensitivity analyses of the complete-case population and the last observation carried forward supported the results from the ITT analyses (complete cases are described in eTable 4 in Supplement 2). There were no relevant differences between the main analysis and the sensitivity analysis.

## Discussion

The treatment of patients with DMD remains an unmet medical need. Recent therapeutic advances that target specific mutations strongly limit the number of patients who are able to benefit from these treatment options.^31,32^ To explore a new mutation-independent therapy, we investigated the efficacy and safety of treatment with a combination therapy in ambulant patients with DMD.

Although we detected a clinically relevant mean between-group difference of 5.5% (corresponding to a 58% reduction in transfer and standing motor function decline) at 26 weeks in favor of patients treated with combination therapy, this study did not demonstrate a statistically significant treatment benefit. The clinical variability among the participants was larger than we assumed in our sample size estimation; therefore, a larger variability across individual treatment effects was observed. One reason for this uncalculated variability was the improvement of muscle function observed among some young patients (aged 6.5-8 years at baseline) in the unstable subgroup who received placebo. These patients continued to gain muscle function during the study period owing to normal motor development, which resulted in higher interindividual variability and lower statistical significance. In contrast, in the predefined stable subgroup of patients with a baseline 6-MWD of 350 m or more, a very low variability with a steady muscle function decline was observed among those receiving placebo. In this subgroup, a clinically meaningful and significant reduction of the MFM D1 subscore decrease of 6.7% was observed in patients treated with combination therapy compared with placebo. Furthermore, in the overall population and the stable subgroup, key secondary clinical end points, including MFM total scores, quantitative muscle force assessments, 10-m walk tests, and supine uptimes at weeks 13 and 26, were consistently in favor of the combination therapy group compared with the placebo control group (Figure 2).

Laboratory end points related to muscle necrosis were also consistently better among patients treated with combination therapy (Figure 2). A significant reduction of nonessential amino acids in the urine of patients receiving combination therapy compared with those receiving placebo represents a reduced loss of amino acids in the urine, which is indicative of reduced muscle degeneration. The MRI findings were of special interest for our study. The decrease in muscle degeneration measured by qMRI muscle sequences was significantly in favor of the treatment group compared with the control group. These results highlight a meaningful reduced transformation of normal muscle into fatty tissue by 66% in the treatment group compared with 58% in the control group. To our knowledge, combination therapy is the first to show a significant slowing in muscle degeneration based on an analysis of qMRI results in a randomized placebo-controlled clinical trial. Because corticosteroids slow muscle degeneration, as assessed by qMRI,^33^ patients with DMD who were treated with corticosteroids demonstrated a smaller increase in FF after 1 year of treatment compared with age-matched patients who were not treated with corticosteroids. However, in contrast to the results of our study treatment, no significant differences in FF were detectable after 3 and 6 months of corticosteroid treatment.^33^

Overall, the significant differences in the MFM D1 subscores of the larger and more homogeneous stable subgroup, the increasing differences between the treatment and control groups over time, the significant findings from the qMRI examinations, and the consistent results from the clinical and laboratory end points are supportive of combination therapy for the treatment of patients with DMD; however, these findings also indicate that this study may have lacked sufficient statistical power. The reasons for this lack of power may include the fact that the sample size calculations were based on data from a pilot study of only 5 patients,^18^ and motor function improvements among the young, still-developing patients in the control group were not considered. Furthermore, we were surprised to detect altered baseline NO-related amino acid plasma concentrations, which suggest that DMD may be associated with an increased l-citrulline metabolism in patients. A total of 36% of patients had l-citrulline values below the lower limits of normal (defined as 18-50 μmol/L), and 76% of patients had l-ornithine plasma levels above the upper limit of normal (defined as 24-64 μmol/L). This observation provides further indication that a possible NO-related amino acid disturbance may be associated with muscle dysfunction in patients with DMD.

In this study, treatment with combination therapy was safe and well tolerated. The most common AE was the occurrence of mild, transient gastrointestinal symptoms, which is a known AE of metformin treatment. Severe AEs occurred in only 3 patients in the treatment group; 2 of those were bone fractures, which are common complications in patients with DMD, indicating that an association with the combination therapy is unlikely.

### Limitations

This study was limited by a short observation period of 26 weeks. Other limitations included a small patient sample and the unknown contributions of each individual substance (l-citrulline and metformin) to the observed effects.

## Conclusions

This study found that treatment with combination therapy was not associated with an overall reduction in motor function decline among ambulant patients with DMD; however, a reduction in motor function decline was observed among the stable subgroup of patients treated with combination therapy. Combination therapy has a better safety profile than corticosteroid treatment and is suitable for any patient with DMD, whatever his genetic condition; thus, the administration of combination therapy represents a promising treatment option to ameliorate muscular metabolism and reduce clinical decline in patients with DMD. A larger and longer multicenter study is planned to evaluate whether treatment with combination therapy is associated with a delay in the progression of DMD.

## Notes

Supplement 1. **Trial Protocol**

Supplement 2. **eTable 1.** Change in MFM D1 Subscore, Secondary End Points (Biomarkers Reported Separately), and Subgroup Analysis From Baseline to Week 26 in the Complete-Case Population **eTable 2.** Overall Study Results Week 13 in the ITT Population **eTable 3.** Changes in Biomarkers Week 26 in the ITT Population **eTable 4.** Changes in Biomarkers Week 13 in the ITT Population **eFigure.** Changes in Mean Motor Function Measure Subscore D1 at Weeks 13 and 26 Compared to Baseline

Supplement 3. **Data Sharing Statement**
