# Supplementary material for: Effect of Combination l-Citrulline and Metformin Treatment on Motor Function in Patients With Duchenne Muscular Dystrophy: A Randomized Clinical Trial
Source: JAMA Netw Open. 2019 Oct 30;2(10):e1914171. doi: 10.1001/jamanetworkopen.2019.14171 (PMC6824222; doi:10.1001/jamanetworkopen.2019.14171)
Supplement: Supplement 1. — Trial Protocol [file jamanetwopen-2-e1914171-s001.pdf]

TRIAL PROTOCOL

1. TITLE OF THE PROTOCOL

"A double blind randomised placebo controlled efficacy and safety study of L-citrulline and metformin in ambulant children aged between 6.5 and 10 years with Duchenne's muscular dystrophy"

Version 8, September 11th, 2015

Investigator:
Name/Title: PD Dr. med. Dirk Fischer
Division/Hospital: Neuropaediatrics, UKBB and Neurology, University Hospital, Basel

Signature: ………………………………………….. Date: ………………………….

List of employees:

Daniela Rubino, study coordinator
Dr. med. Ulrike Bonati (from 04/01/2013), Co-investigator

2. BACKGROUND INFORMATION

2.1 Description and indication of the trial products

L-citrulline

L-citrulline is a non-essential amino acid not coded for by DNA, and in that sense, it is not involved in protein synthesis. However, L-citrulline forms a central part of the urea cycle. As precursor of L-arginine it is transformed into nitric oxide (NO) by nitric oxide synthase (NOS). Intake of L-citrulline in MELAS (Mitochondrial encephalomyopathy, lactic acidosis, and stroke-like episodes) patients increased L-arginine and NO plasma concentration of, substantially higher than the same dose of L-arginine (1). Therefore, we use L-citrulline instead of L-arginine. In addition, L-citrulline is not metabolised in the gastrointestinal tract thereby not leading to gastrointestinal side effects that sometimes can be observed with L-arginine.

Metformin

Metformin is an oral biguanide anti-diabetic drug approved by Swissmedic for the treatment of an increased insulin resistance and therapy of type 2 diabetes. In the EU and in Switzerland it is also approved since 2004 for this indication in children starting from the age of 10 years (2). The blood glucose lowering effect of metformin is based on an effect which breaks the insulin resistance in liver and muscle. The safety and efficacy of the use of metformin in children and adolescents have been demonstrated before the market authorisation in a randomised double-blind study with 82 children and adolescents aged between 10-16 years. Recent data in 53 insulin resistant children aged between 6-12 years (thereof 19 aged between 6-10 years) showed a good safety profile (3). Metformin does not have a stimulating effect on the insulin secretion and does on its own not lead to hypoglycaemia.

2.2 Reason for the dosage and dosage plan

L-citrulline

The normal values of the plasma concentration of L-arginine in children aged between 8-11 years are 54,29 to 115,33 µmol/l. By daily oral intake of L-arginine 3 x 2.5 g d a doubling of the plasma concentration in children aged between 7-17 years was observed (4). This also corresponds to the dose of 3 x 0.3-0.5 mg/kg/d which was used to increase the plasma concentration of L-arginine >100 µmol/l in patients with MELAS (5). Single doses up to 15 g of

L-citrulline have been tolerated without side effects (6). Therefore, in this study all actively treated patients will get a daily dose of 3x 2.5g L-citrulline, , respectively placebo (provided in single sachets which have to be dissolved in a glass of water or fruit juice) per day during 26 weeks. L-citrulline sachets have to be stored at room temperature (15-25°C).

Metformin

For the planned study a dosing of 250 mg metformin, respectively placebo, three times a day (750 mg daily) will be given during 26 weeks. In our mentioned recent pilot study we used 2 x 250 mg/d metformin and observed a very good safety profile, no serious side effects, and no drop-outs. To increase the efficacy and facilitate the dosage regimen, we want to increase the metformin dose and provide it also at a three times daily dosage (750 mg daily). A dose up to 2000 mg/d seems to be associated with a low incidence of gastrointestinal side effects in children (7) thus a dose of 750 mg/d seems to be safe and might even more activate the muscle metabolism. At the beginning of the treatment with metformin nausea, vomiting, diarrhoea and abdominal pain may occur. Usually these symptoms decrease spontaneously after a few days.
To ensure the blinding, metformin capsules produced by the hospital pharmacy of the University Hospital Basel will be used in this trial. They have to be stored at room temperature (15-25°C).

3. OBJECTIVES AND PURPOSE

3.1 Background, reason for the study and objective of the study

Duchenne muscular dystrophy (DMD) is an X-linked recessive disease and it is the most common inherited muscle disorder. DMD is characterized by replacement of normal muscle tissue by connective and fatty tissue resulting in progressive weakness with early loss of free ambulation. At the age of 3-4 years patients first present with muscle weakness due to an irreversible, progressive loss of skeletal muscle, which confines patients to a wheelchair by about 10 years of age. Typically, death occurs around 25 years of age due to cardio-respiratory complications. Current therapeutic management is supportive (8,9).
In muscle the disease causing gene product, dystrophin, is located at the inner surface of the plasmalemma. It interacts both with a number of membrane proteins that form the dystrophin-associated glycoprotein complex (DGC) and the intracellular cytoskeletal proteins. Loss of dystrophin in DMD is associated with a loss of cytoskeletal integrity. This structural defect gives

rise to a misregulation of calcium ions and activation of proteases such as calcium-dependent neutral proteases (calpains). The loss of DGC proteins also results in a severe reduction of neuronal nitric oxide (NO) synthase (nNOS) in DMD (10). In normal subjects, nNOS activity leads to intramuscular L-arginine degradation to NO. Arginase II (that competes with nNOS) degrades L-arginine to L-ornithine and is upregulated in DMD (11). NO concentrations have not been measured in DMD, but the reduced nNOS activity and increased arginase II activity in DMD suggest a lower NO concentration in DMD muscle compared to controls (Fig 1a, b). NO stimulates upregulation of nuclear genes involved in mitochondrial biogenesis, including SIRT1 and PGC-1α (12,13). In addition, NO activates AMP-activated protein kinase (AMPK), which is a critical regulator of muscular energy balance. AMPK stimulates fatty acid uptake, increases fatty acid oxidation, and mitochondrial function and biogenesis. AMPK exerts its action partially via nNOS phosphorylation (14). Thus, NO and AMPK have both been shown to increase mitochondrial function and biogenesis in a synergistic mode of action, but both also act independently.

In DMD an increased production of reactive oxygen species (ROS) has been observed, which can cause further protein and membrane damage (15). The major sources of intracellular ROS are mitochondria, implying an altered mitochondrial function in DMD. Indeed, decreased mitochondrial oxidation rates in muscle biopsies from DMD patients were reported by Sperl et al (16). In accordance to an impaired mitochondrial function a low fat utilization at an early stage of the disease has been suggested and muscle tissue is increasingly being replaced by fatty tissue (17,18). Therefore, we suggest that increasing NO concentrations seems to be promising to ameliorate the devastating effects in DMD via stimulation of mitochondrial function, improved fat utilisation, and energy production (Fig. 1c). Indeed, direct NO donors can improve prednisone effects on muscular dystrophy in the mdx mouse diaphragm (19). L-arginine treatment also improved functional abilities of mdx mice (20). In addition expression of a muscle-specific, nNOS transgene increases the endurance of mdx mice during treadmill running (21).

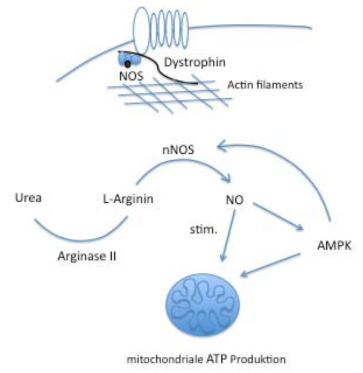

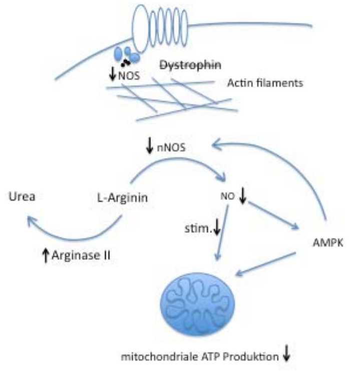

a) normal muscle cell

b) DMD, untreated

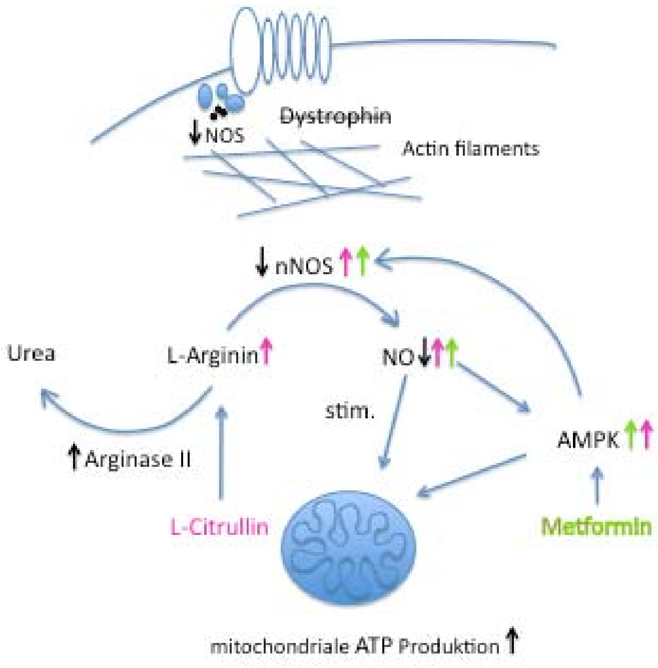

c) DMD, suspected changes when treated

Figure 1: Nitric oxide metabolism in healthy (a) and DMD (b) muscle. (C) showing the suspected and the proposed approach to overcome the NO and energy deficit in DMD muscle.

3.1.1 Own pilot study

After having established all necessary assessment tools in Basel we performed a small pilot study to examine if a 16 week treatment with 3 x 2,5 g/d of L-arginine (NO precursor) and 2 x 250 mg/d Metformin (pharmacological AMPK activator) could serve as therapeutic treatment for DMD. All necessary permissions from the local ethical committee and the Swissmedic were obtained before the beginning of the study. A total of five ambulant DMD patients aged between 7-10 years were enrolled and treated in our pilot-study. One patient was treated with corticosteroids (standard treatment of care), while others had refused to take steroids because of possible side effects before they entered the trial. No major side effects were observed. No patient dropped out. In addition to the presented data (see below) we performed muscle biopsies. We are currently analysing the gene expression in muscle tissue of proteins (baseline and after treatment) involved with NO metabolism, reactive oxidative stress, fatty acid oxidation, and mitochondrial biogenesis.

Laboratory testing revealed no significant change in creatine kinase levels, renal and liver function tests, glucose, and HDL / LDL values (baseline vs. posttreatment). DEXA scans revealed that the average whole body muscle content (70,6 % baseline vs. 70,3% posttreatment) and fat content did not change significantly. Indirect calorimetry assessing energy and muscle metabolism in vivo showed important changes. As previously postulated metabolism changed and the relative carbohydrate contribution to the oxidation rate decreased in all patients (mean change -17,9%), while the relative fatty acid contribution to the oxidation rate increased (mean change 13,9%). Quantitative muscle MRI showed that metformin and l-arginine treated patients had an MFC increase of 1,4 % corresponding to an annual increase of 4,4 %. Ambulant DMD patients older than 8 years receiving standard treatment with steroids show a median annual MFC increase of 8-9 % (22). This indicates that L-arginine and metformin may slow the progression of muscle degeneration in DMD.

Due to the progressive nature of DMD MFM values and 2MWD decrease over time until free ambulation is lost (mean progression can be calculated based on data from the literature and correspond to predicted values (23, 24). In contrast, four of the five treated patients showed a marked improvement of their clinical and functional abilities. The mean total MFM (3,5%) and 2MWD (10 meter) improved. In addition, the MFM D1 subdomain (standing and transfers), which is the most informative dimension in ambulant DMD patients aged 6 years and over with a

mean decrease of 17.2% per year (23), improved by more than 6% in our treated DMD group. More importantly, this improvement is better than the maximal mean improvement reported for the total MFM and the D1 MFM subscore (both below 2%) after beginning a steroid treatment (standard symptomatic treatment of care)(25). Relevant changes observed in our trial are shown in table 2.

Category	Variable	mean	median	SD	range	95% CI
calorimetry	REE, kcal/24 h	-51.2	-56.5	37	-84 to -8	-54 to -48.5
calorimetry	carbohydrate oxidation, %	-17.9	-11.3	18.4	-44.8 to -4.4	-20.7 to -15.1
calorimetry	fatty acid oxidation, %	13.9	9.6	13.7	2.9 to 33.6	11.1 to 16.7
Walking	2 min walking distance	9.58	9.6	29.3	-40.3 to 30.6	6.8 to 12.4
MFM	total score, %	3.54	7.29	6.93	-8.33 to 8.33	0.969 to 6.11
MFM	D1 subscore, %	6.15	7.69	11.6	-12.8 to 15.4	3.58 to 8.72
MFM	D2 subscore, %	1.67	2.78	4.65	-5.56 to 5.56	-0.903 to 4.24
MFM	D3 subscore, %	1.91	0	5.43	-4.76 to 9.53	-0.665 to 4.48

Table 1: Descriptive statistics (mean, median, standard deviation, range, and 95% confidence interval) for selected variables of all five treated patients (change from baseline to post-treatment).

Individual and predicted (calculated using data from the literature (23,24)) MFM and the 2 min walking distance values of all treated patients are presented as follows:

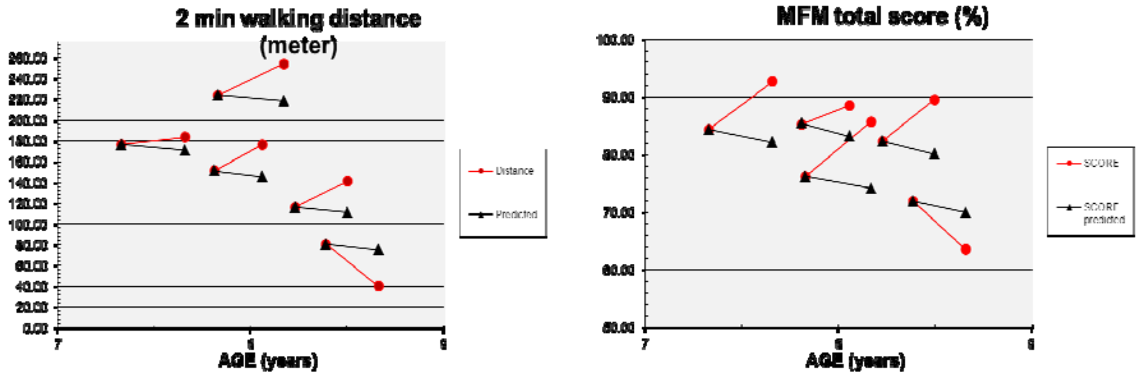

Figure 2: Individual and predicted (calculated using data from the literature (23,24)) 2 min walking distance (A) and MFM (B) values of all treated patients.

3.2 Questionnaire, trial population

Our aim is to confirm in a double blind placebo controlled trial the observed changes of our own 16 week pilot study in a broader population being treated as postulated by current medical guidelines, including patients under steroid treatment. The precursor study performed on 5 ambulatory DMD patients that showed an improved lipid metabolism, improved functional abilities, prolonged walking distances, and slowing of the rate of progressive fatty degeneration of involved muscle observed on quantitative MRI.

We plan to perform a 26 week placebo controlled RCT (randomized controlled trial) with a total number of 40-50 ambulant DMD patients aged between 6.5 and 10 years. If L-citrulline and metformin are helpful to improve muscle function, a better symptomatic treatment and slowing of muscle degeneration in DMD could be expected, other multicenter trial will be performed.

To obtain normal values for measures of muscle metabolism and mitochondrial function blood and urine samples will be analysed in 10 – 20 healthy age matched male children and 10 – 20 healthy male controls.

3.3 Hypothesis

Combined both causes, the increased reduction of L-arginine by arginase II and the decreased NO production by reduced nNOS should be treated to enhance the NO concentration and thus to achieve an improvement of muscular strength, a deceleration of muscle atrophy and long-term an extension of time until loss of ambulation.

4. DESIGN OF THE TRIAL

4.1 Targeted primary endpoint and secondary endpoints

<u>Primary outcome measure</u>

1) Mean change of MFM D1 subscore from baseline to week 26 under L-citrulline/metformin therapy compared to placebo

Several tests have been reported in the literature to assess muscle strength and functional ability, to monitor the progression of the disease, and to evaluate the results of drug interventions and rehabilitation. However, most instruments involve only ambulant patients, making adjustments and/or additional assessments necessary when the disease progresses. The MFM, a validated assessment tool to measure motor function in both ambulant and non-ambulant patients with neuromuscular disorders, was developed in 2005 in France. It includes 32 items that evaluate three dimensions of motor performance, including specific motor functions, such as transfers and standing posture (D1), proximal and axial (D2), distal (D3) and a total MFM score involving all of the motor dimensions. The items are scored and summed to comprise a total score, in which the maximum represents normal motor function. The instruction manual, validation examinations and other publications using the MFM can be downloaded at the MFM website (27). In a recent work in DMD, the annual decrease ot the total score was 5.8% for the whole group of DMD patients, indicating an overall decline in motor capacities. However, examination of the D1, D2, and D3 subscores independently provided more information depending on the stage of the disease. Indeed, in ambulant patients with DMD, D1 was the most informative dimension, with a mean decrease of 17.2% per year before loss of ambulation in patients aged 6 years and over. After loss of ambulation, D2 became the most informative and showed an average decrease of 9.4% per year. In patients over 14 years, the average decrease in D3 was 10.8% per year (23). D1 seems to us to be particularly interesting because it is related to loss of ambulation and is responsive to short-term changes (3 months) and should provide sufficient information in a 6 months trial. Furthermore, in our own 16 week L-arginine and metformin pilot trial (see below 2.2.4) in ambulant DMD patients the D1 subscore showed the best effect size of all evaluated clinical parameters. Timed clinical functional assessments included the 2 min walking distance in meter (2MWD). In addition to the MFM timed clinical functional assessments (as the 6 min or 2 min walking distance in meter (6MWD /2MWD)) are helpful to assess the muscle function in children with DMD. Due to the progressive nature of DMD MFM values and 2MWD over time until free ambulation is lost (mean progression can be calculated based on data from the literature and correspond to predicted values) (24,25). The 6MWD is also a validated tool to measure the distance an individual is able to walk over a total of six minutes on a hard, flat surface. The goal for the individual is to walk as far as possible in six minutes. The individual is allowed to self-pace and rest as needed as they traverse back and forth along a marked walkway. Data from 112

DMD patients aged 4–17 years in the Italian cohort showed that the 6MWD performances increased (approximately 33 m/year) with age up to 7 years, with a clear point of slope change at approximately 7 years. After the age of 7 years, there was a variable decline in the 6MWT (-12 m/year) (24).

<u>Secondary outcome measures:</u>

2) Mean change of MFM total score, the D2, and D3 MFM subscores from baseline to week 26 under L-citrulline/metformin therapy compared to placebo (for details see 1)

3) Mean change of walking distance from baseline to week 26 under L-citrulline/metformin therapy compared to placebo assessed with the six-minute walk test (for details see 1)

4) Change of quantitative muscle MRI including muscle fat content (MFC) and T2 times of thigh muscles visualized by MRI (Magnetic Resonance Imaging) in both groups at week 26 versus baseline (for details see 2.2.2)

5) Change in the plasma / urine concentration for markers of muscle necrosis (resting creatine kinase, plasma), oxidative stress (8OHDG, urine; carbonyliated proteins, 4-HNE, serum), nitrosative stress (nitrotyrosin, plasma; ADMA, serum; cGMP, urine), and change of miRNAs serum concentration in both groups at week 26 compared to baseline and compared to healthy controls.

6) Mean change of quantitative muscle force (QMT) of knee extension and elbow flexion using hand held dynamometry (HHD) from baseline to week 26 under L-citrulline/metformin therapy compared to placebo

Aims of our treatment are a stimulation of NO concentrations (indirect markers nitrotyrosine, ADMA and cGMP), an improvement of the muscular energy situation with reduction of oxidative stress (8OHDG, carbonyliated proteins, 4-HNE as markers) and a slowing of the

muscle denegeration (creatine kinase as marker.) Aim of our treatment is a slowing of the natural history of the disease, so we plan to analyse possible serum biomarkers (miRNAs) of disease progression (26).

4.2 Trial design

This is a randomised placebo controlled trial. We plan to enrol 40-50 ambulant DMD patients aged between 6.5 and 10 years. All actively treated patients will get a daily dose of L-citrulline of 7.5 g/d separated in three doses per day (3x 2.5 g) as well as metformin 250 mg three times a day (750 mg daily). The patients randomized to the placebo group will receive matching placebo (sachets and powder and capsule look like verum but do not contain active ingredient). Treatment is given for the period of 26 weeks. Co-medication with glucocorticoids (standard treatment of care) is allowed. At baseline as well as at the end of the study clinical measures, laboratory, and MRI measures will be performed. This includes the MFM scale; 6MWT, MFC assessment using quantitative thigh muscle MRI, and laboratory blood analysis.

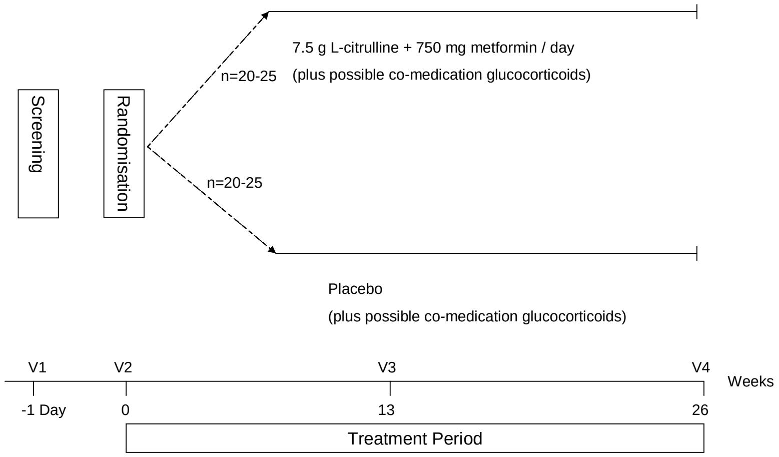

Figure 3: Flow chart showing the study design with screening, randomisation, and treatment period.

	**Visit 1**	**Visit 2**	**Visit 3**	**Visit 4**
	**Screening (Day -1)**	**Baseline (week 0)**	**Week 13** ±7 days	**End of Study (week 26)** ±7 days
Patient Information/Informed Consent	x			
Inclusion/Exclusion	x	x		
Vital signs (blood pressure, heart rate, weight, height)	x	x	x	x
Physical examination (general and neurological)	x	x	x	x
MFM scale	x*		x	x
muscle force of knee extension and elbow flexion using hand held dynamometry				
6MWT	x*		x	x
10 m within 10 s walking test	x*		x	x
MRI		x		x
Capture AEs		x	x	x
Blood and urine analysis: CK, amino acids, miRNAs, haematology[1], chemistry[2], markers of oxidative stress[3] and NO function[4]		x	x	x
metformin serum concentration (compliance check)				x
Randomisation		x		
Dispensing of study medication		x	x	
Collection of study medication			x	x

Table 2: Planned visiting and examination schedule.

* will be used as baseline values

[1] Full blood count: erythrocytes, reticulocytes, leucocytes, platelets, haemoglobin, haematocrit
[2] GOT, GPT, creatinine, electrolytes (Na, K, Ca), urea, CK, HbA1c, cholesterol, HDL, LDL, triglycerides
[3] 8OHDG (urine), carbonyliated proteins (serum), 4-HNE (serum)
[4] Nitro-tyrosin (serum), ADMA (serum), cGMP (urine)

Visit 1 = Screening (Day -1)

After signing the informed consent form the inclusion and exclusion criteria are verified. If the criteria are fulfilled the patient will be enrolled in the study.

During this visit the following procedures will be performed:

- Vital signs
- Physical examination
- MFM scale
- 6MWT
- muscle force of knee extension and elbow flexion using hand held dynamometry
- 10 m within 10 s walking test

Visit 2 = Baseline (Week 0)

During this visit the following procedures will be performed:

- check inclusion/exclusion criteria
- vital signs
- physical examination
- MRI
- Adverse events
- laboratory
- dispensing of study medication

If the patient still qualifies for the study, he/she will be randomized and receive study medication.

Visit 3 (Week 13, ±7 days)

During this visit the following procedures will be performed:

- vital signs
- physical examination
- MFM scale
- 6MWT
- muscle force of knee extension and elbow flexion using hand held dynamometry
- 10 m within 10 s walking test

- Adverse events
- laboratory
- dispensing of study medication
- collection of study medication

Visit 4 = End of Study Visit (Week 26, ±7 days)

During this visit the following procedures will be performed:

- vital signs
- physical examination
- MFM scale
- 6MWT
- muscle force of knee extension and elbow flexion using hand held dynamometry
- 10 m within 10 s walking test
- MRI
- Adverse events
- laboratory
- collection of study medication

Patients (or their parents) can voluntarily withdraw from the study at any time. However, the investigator should try to assess the main reason and capture this information in the CRF.

In case of screening failure due to exclusion criteria the patient can be re-screened once for this study.

4.2.1 Healthy volunteers

10–20 age matched healthy boys within the same age range and 10 – 20 healthy male controls will be enrolled to obtain normal values of mitochondrial and muscle metabolism. All healthy controls will be seen at two time points (screening and for blood drawing). Healthy children (and their caregivers) will be asked to participate in our study while undergoing an elective surgery at our hospital. By this blood drawing will be done as part of the routine safety protocol while the child is already under anaesthesia. An additional amount of 4 ml of blood will be drawn for this study but no additional venous puncture will be necessary for this study.

Healthy adult controls will be identified for potential recruitment using IEC/IRB approved newspaper advertisements, mailing lists, websites or database available at UKBB/USB.

Visit 1 = Screening

After signing the informed consent form the inclusion and exclusion criteria are verified. If the criteria are fulfilled the patient will be enrolled in the study.

Visit 2 = Baseline

During this visit the following procedures will be performed:

- check inclusion/exclusion criteria
- blood drawing

4.2.2 Criteria for stopping the study

The following conditions/events can lead to an early termination of the study:

- withdrawal of consent
- protocol violations caused by the patients (noncompliance)
- logistical reasons (relocation of patient etc.)
- circumstances which do not permit any more regular visits in the context of the study
- abnormal laboratory values, including liver or renal function tests (transaminasis >2 x ULN, creatinine >2 x ULN)
- abnormal increase in blood pressure as determined by the investigator
- For safety of the patient as considered by the investigator

4.2.3 Conditions for unblinding

Unblinding is the process by which the allocation code is broken so that the investigator, clinical staff and/or the trial statistician becomes aware of the intervention for a person participating in a trial. The usual reason for unblinding is that a person participating in the study has encountered an urgent medical problem necessitating that the clinician know his/her intervention allocation.

The principal investigator assess the need for unblinding where a serious adverse event has occurred and the treatment or allocation code is required in order to enable clinical treatments to

be planned. Upon unblinding, the site personnel will record the participant withdrawal and allocation in the person's clinical and trial notes along with the appropriate clinical notations.
Unblinding envelopes**:** A series of envelopes, each labelled with the randomisation number that contain the allocation for that person. These envelopes are sealed by the central randomisation service, and are only opened if emergency unblinding is required.

4.3 Measure to minimise bias (randomising, blinding)

Patients who meet the study admission criteria will be enrolled in the study and a single subject identification number will be assigned. Patients are allocated to the two study groups, L-citrulline and metformin or placebo, in a 1:1 ratio. The assigned number will be recorded in the CRF Visit 2. Drop outs after baseline visit (randomisation) will not be replaced.
The physiotherapists who perform the tests were trained and certified in Lyon where MFM has been established and validated.
This is a double blind, placebo controlled, randomized study.

5. SELECTION OF TRIAL SUBJECTS

5.1 Recruitment

The study patients will be recruited among the affected children in the neurologic clinic of the UKBB as well as using the Swiss and German DMD Register. Currently approximately 120 DMD patients are registered in the Swiss Register. To find the patients the Swiss DMD Register will be searched in alphabetic, systematic order for inclusion and exclusion criteria and matching patients will be contacted and informed about the study. Furthermore, an advertisement will be placed on the website Swiss and German DMD Register after approval of the Ethics Committee and Swissmedic.
Healthy volunteers will be recruited from our hospital. Children and their caregivers having a routine blood drawing prior to an elective surgical procedure will be asked if they are willing to give an additional amount of blood for this study.

5.2 Inclusion criteria

- Molecular diagnosis of DMD

- Patients 6.5 - 10 years of age at time of screening
- Ambulant
- Ability to walk 150 m in the 6 min walking distance (6MWT)
- D1 subdomain of the MFM scale >40%
- stable treatment with steroids for >6 months or steroid naïve patients

5.3 Exclusion criteria

- Previous (3 months or less) or concomitant participation in any other therapeutic trial
- Use of L-citrulline, L-arginine or metformin within the last 3 months
- Known individual hypersensitivity to L-citrulline or metformin
- known or suspected malignancy
- Other chronic disease or clinical relevant limitation of renal, liver, heart function according to discretion of investigator
- start of cortisone treatment or change in dosage <6 months prior to screening

5.4 Inclusion criteria healthy volunteers

- Male sex
- Aged between 6.5-10 and 18-60 years.

5.5 Exclusion criteria healthy volunteers

- Previous (3 months or less) or concomitant participation in any other therapeutic trial
- Use of L-citrulline, L-arginine or metformin within the last 3 months
- Known genetic or acquired neuromuscular disorder
- known or suspected malignancy
- Other chronic disease or clinical relevant limitation of renal, liver, heart function according to discretion of investigator

6. ASSESSMENT OF EFFECTIVENESS

6.1. Effectiveness parameter: measuring methods and time

1) Mean change of MFM D1 subscore from baseline to week 26 under L-citrulline/metformin therapy compared to placebo

2) Mean change of MFM total score, the D2, and D3 MFM subscores from baseline to week 26 under L-citrulline/metformin therapy compared to placebo (for details see 1)

3) Mean change of walking distance from baseline to week 26 under L-citrulline/metformin therapy compared to placebo assessed with the six-minute walk test (for details see 1)

4) Change of quantitative muscle MRI including muscle fat content (MFC) and T2 times of thigh muscles visualized by MRI (Magnetic Resonance Imaging) in both groups at week 26 versus baseline (for details see 2.2.2)

5) Change in the plasma / urine concentration for markers of muscle necrosis (resting creatine kinase, plasma), oxidative stress (8OHDG, urine; carbonyliated proteins, 4-HNE, serum), nitrosative stress (nitrotyrosin, plasma; ADMA , serum; cGMP, urine), and change of miRNAs serum concentration in both groups at week 26 compared to baseline.

6) Normal values for makers of oxidative stress, nitrosative stress and changes of mi-RNA in serum and urine will be obtained in 10-20 healthy male adults aged 18 to 60 years and 10-20 boys aged 6.5 to 10 years.

6) Mean change of quantitative muscle force (QMT) of knee extension and elbow flexion using hand held dynamometry (HHD) from baseline to week 26 under L-citrulline/metformin therapy compared to placebo

7. SAFETY

7.1 Safety parameter: measuring methods and time

At every study visit a clinical examination will be performed and the vital parameters blood pressure and heart rate will be measured. The assessment of blood chemistry and of the following parameters is foreseen at every visit (respectively visit 1 or 2):

- full blood count, as with the intake of metformin in the context of the registration trials isolated cases of leucopoenia, thrombopenia, and haemolytic anaemia were observed
- clinical chemistry (transaminasis, creatinine, electrolytes, urea),
- marker of the muscle necrosis (creatine kinase concentration),
- makers of oxidative stress: (8OHDG urine concentrations, carbonyliated preoteins and 4-HNE serum concentration)
- markers of nitrosative stress (nitrotyrosin, plasma; ADMA; serum; cGMP, urine)
- all amino acids
- glucose metabolism marker (HbA1c)
- lipid metabolism marker (cholesterol, HDL, LDL, triglycerides)

The intake of metformin has to be stopped in case of clinically significant changes (increase of creatinine and transaminasis >2 x ULN)

If pathologic changes independent of the known muscle disease should be detected, the affected patients will be informed immediately and the possibilities of further investigation, respectively treatment of these abnormalities according to current medical knowledge will be discussed.

7.2 Follow-up observation for trial subjects with adverse reactions

Patients with adverse reactions which have occurred in the context of the study will be followed up by the investigator up to 30 days after the last visit.

8. STATISTICS

8.1 Definition of the primary end point and secondary endpoints

1) Mean change of MFM D1 subscore from baseline to week 26 under L-citrulline/metformin therapy compared to placebo

2) Mean change of MFM total score, the D2, and D3 MFM subscores from baseline to week 26 under L-citrulline/metformin therapy compared to placebo

3) Mean change of walking distance from baseline to week 26 under L-citrulline/metformin therapy compared to placebo assessed with the six-minute walk test

4) Change of quantitative muscle MRI including muscle fat content (MFC) and T2 times of thigh muscles visualized by MRI (Magnetic Resonance Imaging) in both groups at week 26 versus baseline

5) Change in the plasma / urine concentration for markers of muscle necrosis (resting creatine kinase, plasma), oxidative stress (8OHDG, urine; carbonyliated preoteins, 4-HNE serum), nitrosative stress (nitrotyrosin, plasma; ADMA, serum; cGMP, urine), and change of miRNAs serum concentration in both groups at week 26 compared to baseline values and to values of age and sex matched healthy volunteers.

6) Mean change of quantitative muscle force (QMT) of knee extension and elbow flexion using hand held dynamometry (HHD) from baseline to week 26 under L-citrulline/metformin therapy compared to placebo

8.2 Planned number of trial subjects with clearly stated justification (possibly power analysis)

For sample size estimation the MFM D1 subscore was chosen. Baseline and post-treatment measurements of 5 patients are available from the pilot study. A semi-parametric approach for sample size estimation was chosen that made use of these data. For the 2 min. walking distance, a mean change from baseline to post- treatment of 10 m was expected (estimated from pilot data). For the MFM D1 subscore, a mean change from baseline to post-treatment of 6.1 % was expected (estimated from pilot data). Sample size estimation was repeated for a range of values smaller and larger than the expected mean changes. Further, the influence of two randomisation ratios for the verum/placebo groups, 1:1 and 2:1, on sample size was checked. Each sample size, $n_{i=1,...,40}$ = 11, ..., 50 for the MFM D1 subscore, was evaluated by drawing 99 times an individual data set of size n_i from the pilot study data set (sampling with replacement). In each of these individual data sets, each patient was randomly assigned to a verum or a placebo group. For each patient, the post-treatment value was

calculated as the sum of the baseline value and a random variate drawn from a normal distribution with mean and standard deviation estimated from the pilot data. In the case of the placebo group, the mean was set to $-17.2/54 \times 16$ for the MFM D1 subscore (value taken from Table 2 in (39)). (The values from the literature refer to changes within one year, i.e. 54 weeks, and the pilot study lasted 16 weeks, thus the linear transformation of the values by $/\ 54 \times 16$.). For the primary endpoint candidate (MFM D1 subscore), assuming a drop-out rate of 10 %, a randomisation ratio of 1:1, and a power of 0.8, c. 42 patients should be recruited in order to be able to analyse 38 complete datasets. Fig. 4 shows how the sample size behaves with regard to the MFM D1 subscore, the power, and the randomisation ratio.

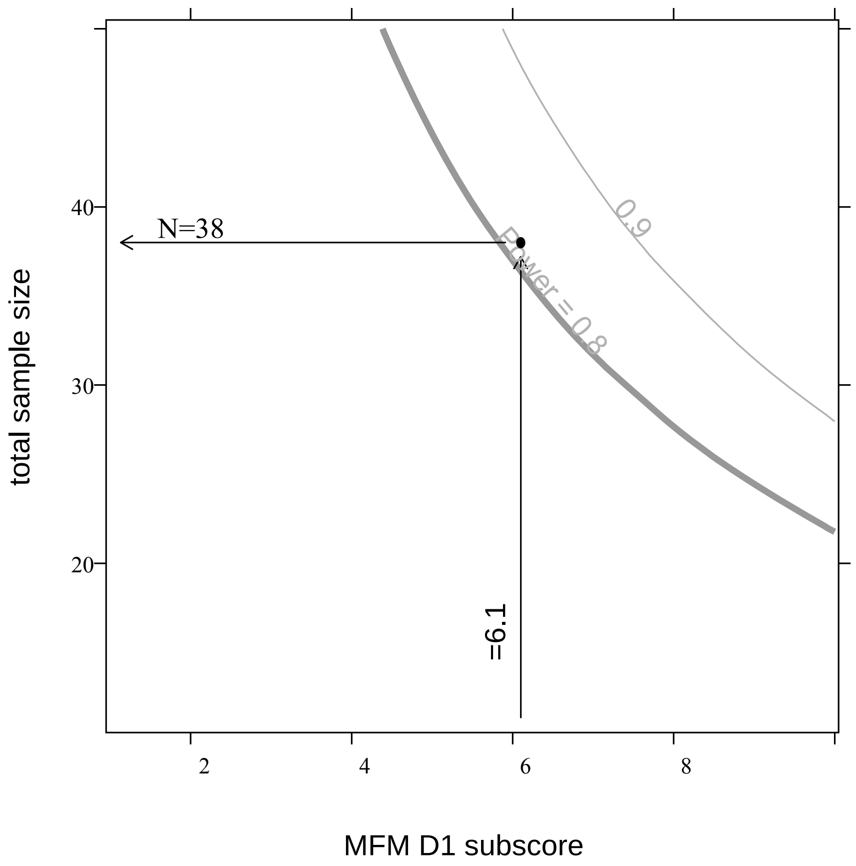

Fig. 4. Sample size with regard to the MFM D1 subscore and the power for a randomisation ratio of 1:1. The numbers on the curves indicate the power. The arrow shows how to interpret the plot. The curves are smoothed and only serve illustrative purposes.

8.3 Description of the statistical methods foreseen and the planned intermediate assessments

Non-parametric Wilcoxon signed rank test

8.4 Planned significance level

$\alpha = 0.05$

8.5 Handling of missing data or of data in the case of subjects halting the trial prematurely

Last observation carried forward

8.6 Definition of the evaluation groups

Intention-to-treat (ITT) analysis

9. TRIAL-SPECIFIC PREVENTIVE MEASURES AND DUTIES

9.1 Trial-specific preventive measures and duties

Trial-specific preventive measures and duties are not necessary.

9.2 Final examination in case of premature withdrawal from the trial

In this case it is necessary to perform a final medical examination for the safety of the child (patient group). The following procedures will be performed:

- vital signs
- physical examination
- MFM scale, 6MWT, 10 m within 10 s walking test, muscle force of knee extension and elbow flexion using hand held dynamometry
- MRI
- Adverse events
- laboratory
- collection of study medication

10. DUTIES ON THE PART OF THE INVESTIGATOR

10.1. Study conduct

The study will be conducted according to the protocol, GCP and the legal requirements as required by Swiss law.

All changes of protocol as well as a final report will be submitted to the Ethics Committee and Swissmedic.

10.2 Pharmacovigilance

10.2.1. Defining Adverse Events

An adverse event (AE) is any untoward medical occurrence in a patient during or following administration of an investigational product and which does not necessarily have a causal relationship with treatment. An AE can therefore be any unfavourable and unintended sign (including an abnormal laboratory finding), symptom, or disease temporarily associated with the use of the trial drugs, whether or not considered related to the trial drugs.

10.2.2. Defining Serious Adverse Events (SAEs)

A Serious Adverse Event is defined in general as an untoward (unfavourable) event, associated with trial drug or trial procedure, which:

- is fatal. Death may occur as a result of the basic disease process. Nevertheless, all deaths occurring until the last administration of the study agent must be treated as an SAE and reported as such.
- is life-threatening
- requires or prolongs hospitalisation
- results in persistent or significant disability or incapacity
- is a congenital anomaly or a birth defect, or
- may require medical or surgical intervention to prevent one of the outcomes listed above
- Any other significant clinical event, not falling into any of the criteria above, but which in the opinion of the investigator requires reporting.

10.2.3. Defining Suspected Unexpected Serious Adverse Reactions (SUSARs)

All SAEs assigned by the local investigator as both suspected to be related to the trial drugs and unexpected are subject to expedited reporting. An event is unexpected when information is not

consistent with the available product information or investigator brochure, or if they add significant information on the specificity or severity of an expected reaction.

10.2.4. Reporting AEs

AEs will be collected for all patients from screening until 30 days after the last dose of treatment with a protocol IMP.

Information about AEs, whether volunteered by the patient, discovered by the investigator questioning or detected through physical examination, laboratory test or other investigation will be collected and recorded in the study files.

If requested, details of collected AEs will be made available after completion of the study.

10.2.5. Reporting SAEs

SAEs will be collected for all patients beginning with informed consent. SAEs resulting in death or Serious Adverse Drug Reaction (fatal or life-threatening) have to be reported to the EC within 7 calendar days of the PI (or his research team) being informed of the event.

10.2.6. Reporting SUSARs

All SAEs assigned by the local investigator as both suspected to be related to study protocol (treatment/procedures) and unexpected (see definition in section 10.2.3) will be classified as SUSARs and will be subject to expedited reporting to concerned ethic committees (EC) and regulatory authorities (RA).

- SUSARs must be reported to the EC / RA within 7 calendar days of the PI (or his research team) being informed of the event, if they result in death or are deemed to be life-threatening.
- Any SUSARs not resulting in death or deemed to be life-threatening must be reported to the EC / RA within 15 calendar days of the PI (or his research team) being informed of the event.

In addition, the sponsor shall collect these events. The information can be aggregated in a line listing of SUSARs which has to be submitted to the EC and the RA annually by the PI, respectively by the sponsor.

10.3 Insurance

The University Children’s Hospital (UKBB) will compensate the patients for damage that occurs within the framework of the clinical trial. For this purpose, the University Children’s Hospital (UKBB) has taken out insurance in favour of the patients with HDI Gerling Industrieversicherung AG, Dufourstrasse 46, 8034 Zürich.

If the patient or the parents observe health problems or other damages they should contact the responsible physician (PD Dr. med. Dirk Fischer). He will undertake the necessary measures.

11. ETHICAL CONSIDERATIONS

11.1 Evaluation of the risk-benefit ratio

Duchenne’s muscular dystrophy is a serious progressive neuromuscular disease with only little symptomatic possibilities of therapy so far. Affected patients mostly die aged between 20 and 30 from a generalised muscle weakness, mostly from cardiorespiratory complications. In our opinion, the infaust prognosis justifies the broad clinical and imaging procedures in this study population.

11.2 Description of why trial subjects requiring particular protection have been included

The Duchenne’s muscular dystrophy becomes manifest from toddlerhood and an early therapy (mainly glucocorticoid therapy and physiotherapy) can delay severe cardiovascular as well as respiratory complications and thus maintain the quality of life longer.

To investigate the efficacy of metformin and L-citrulline on the muscle in children with Duchenne’s muscular dystrophy validly, it is necessary to include subjects requiring particular protection (children between 6.5 and 10 years). At this age changes in the muscle are identifiable, however the muscle mass is mostly maintained and functionally assessed. With the progression of the disease all patients are wheelchair bound why then the (remaining) clinical abilities can only be evaluated to a limited extent.

11.3 Other ethical aspects

The participation in this study is voluntary. If the patient/the parents do not want to participate, they will not experience any disadvantages concerning the further medical treatment. The same applies if the parents and the patient withdraw their consent at a later time point. They have this possibility anytime. A possible withdrawal of consent respectively the withdrawal from the study can occur without giving any reason. In case of withdrawal the data collected until this time point will be used and the samples (blood) collected in the context of the study will be destroyed. In case of withdrawal the patient will undergo a final visit for medical examination for his own safety.

A placebo group is necessary to demonstrate the efficacy of the used combination (L-citrulline and metformin) and to exclude a possible placebo effect.

There is a great potential that positive results of this study could lead to other large multicentre trials. Furthermore, if we can show an effect of L-citrulline and metformin on muscle function, this will be a major break through and mean a better symptomatic treatment of the fatal muscle degeneration in DMD and probably in other muscular dystrophies.

Blood drawing in healthy children will be done as part of a routine safety procedure before an elective surgery. This will be done while children are already under general anaesthesia and children will not suffer any discomfort from the venous puncture. A small additional amount of blood will be obtained for our study at the same time and no additional venous puncture will be necessary.

12. QUALITY CONTROL AND QUALITY ASSURANCE: DESCRIPTION OF MEASURES

12.1 Quality control

To assure the quality of the study conduct and of the data a monitoring of the study will be performed by a person independent of the study (Clinical Trial Unit, University Hospital Basel). All inclusion and exclusion criteria will be checked, if the data have been recorded correctly in the CRF, if the drug accountability is correct and if during the study SAEs have occurred.

12.2 Data protection, archiving and destruction

In this study personal patient data will be captured. This data will be anonymised and is only accessible to experts. The appropriate experts of the sponsor (or their designees) can survey the conduct of the study with monitoring or audits. In case of inspections these experts and also members of the appropriate authorities can get access to the original data. Also the responsible Ethics Committee can get access to the original data. The confidentiality of the data will be strictly protected during the whole study and when performing the mentioned controls. The name of the patient will not be published in no way in reports or publications arisen from the study.

The paper documents will be stored in a lockable room during 10 years in the archive of the UKBB in a dedicated shelf. The investigator records in the medical file that the patient participates in the study.

All data which will be collected in the context of the trial will first be recorded in the medical file. At a later time point the transcription of the data in the CRF will be done. No data will be captured directly in the CRF.

13. PROCEDURE FOR DRUG ACCOUNTABILITY

During the visits 2 and 3 the patients will be given L-citrulline in the vials prepared by the hospital pharmacy as well as metformin capsules 250 mg each or matching placebo. The patient will be asked to return the study medication as well as the empty vials and boxes to every visit. A member of the study team counts and balances the returned tablets and vials and can check the correct intake. This will be captured in an appropriate form.

14. PROCEDURE TO RECORD COMPLIANCE

A qualified person of the study team will check the number of dispensed/taken medication and complete a study specific drug accountability form.

15. DESCRIPTION FORESEEN FOR THE TEST PRODUCT

All study medication will be labelled. The labels will contain the following information: Study number, name of the investigator, for clinical trial, lot number, expiry date, storage conditions, patient number/randomization number, keep out of reach of children.

16. PUBLICATION OF THE RESULTS

The results of the study will be published independent of the results. These will be published in a medical journal.

17. REFERENCES

1 El-Hattab AW, et al. Restoration of impaired nitric oxide production in MELAS syndrome with citrulline and arginine supplementation. Mol Genet Metab. 2012 Apr;105(4):607-14
2 Arzneimittelkompendium der Schweiz 2013
3 Yanovski JA et al. Effects of metformin on body weight and body composition in obese insulin-resistant children: a randomized clinical trial. Diabetes, 2011;60(2):477-85
4 Bennett-Richards et al. Oral L-arginine does not improve endothelial dysfunction in children with chronic renal failure. Kidney Int. 2002 Oct ;62(4) :1372-8
5 Koga et al. MELAS an L-arginine therapy: pathophysiological therapy of stroke-like episodes. Ann N Y Acad Sci. 2010 Jul;1201:104-10.
6 Moinard C, et al. Dose-ranging effects of citrulline administration on plasma amino acids and hormonal patterns in healthy subjects: the Citrudose pharmacokinetic study. Br J Nutr. 2008 ;99(4):855-62.
7 Yanovski JA et al. Effects of metformin on body weight and body composition in obese insulin-resistant children: a randomized clinical trial. Diabetes, 2011;60(2):477-85
8 Moinard C, et al. Dose-ranging effects of citrulline administration on plasma amino acids and hormonal patterns in healthy subjects: the Citrudose pharmacokinetic study. Br J Nutr. 2008 ;99(4):855-62.
9 Bushby K et al. Diagnosis and management of Duchenne muscular dystrophy, part 2: implementation of multidisciplinary care. Lancet Neurol. 2010;9(2):177-89
10 Brenman J.E. et al. Nitric oxide synthase complexed with dystrophin ans absent from skeletal muscle sarcolemma in Duchenne muscular dystrophy Cell. 1995; 82:743–752
11 http://microarray.cnmcresearch.org/pga
12 McConell GK et al. Central role of nitric oxide synthase in AICAR and caffeine-induced mitochondrial biogenesis in L6 myocytes. Appl Physiol. 2010 108(3):589-95
13 Merry TL et al. Downstream mechanism of nitric oxide-mediated skeletal muscle glucose uptake during contraction. Am J Physiol Regul Integr Comp Physiol. 2010, 299(6):R1656-653
14 Hargreaves M. AMPK-mediated regulation of transcription in skeletal muscle Clin Sci (Lond). 2010 Jan 26;118(8):507-18
15 Kuznetsov AV et al. Impaired mitochondrial oxidative phosphorylation in skeletal muscle of the dystrophin-deficient mdx mouse. Mol Cell Biochem. 1998; 183:87-96
16 Sperl W et al. resolution respirometry of permeabilized skeletal muscle fibers in the diagnosis of neuromuscular disorders. Mol Cell Biochem 1997; 174:71-8
17 Hankard R, et al. Resting energy expenditure and energy substrate utilization in children with Duchenne muscular dystrophy. Pediatr Res. 1996 Jul;40(1):29-33.
18 Gaeta M et al. Muscle fat-fraction and mapping in Duchenne muscular dystrophy: evaluation of disease distribution and correlation with clinical assessments. Skeletal Radiol. 2012 Aug;41(8):955-61.
19 Mizunoya W et al. Nitric-oxide donors improve prednisone effects on muscular dystrophy in the mdx mouse diaphragm. Am J Physiol Cell Physiol 2011
20 Archer JD et al. Persistent and improved functional gain in mdx dystrophic mice after treatment with L-arginine and deflazacort. FASEB J. 2006 Apr;20(6):738-40
21 Wehling-Henricks et al. Loss of positive allosteric interactions between neuronal nitric oxide synthase and phosphofructokinase contributes to defects in glycolysis and increased fatigability in muscular dystrophy Hum Mol Genet. 2009; 18(18): 3439–3451
22 Hafner P, Fischmann A et al. Rate of MFC increase in DMD on quantitative muscle MRI. In preparation
23 Bushby K, Connor E. Clinical outcome measures for trials in Duchenne muscular dystrophy: report from International Working Group meetings.Clin Investig (Lond). 2011 Sep;1(9):1217-1235.
24 Mazzone E, et al. Functional changes in Duchenne muscular dystrophy: a 12-month longitudinal cohort study. Neurology. 2011 Jul 19;77(3):250-6. doi: 10.1212/WNL.0b013e318225ab2e. Epub 2011 Jul 6.
25 Silva EC, et al. Motor function measure scale, steroid therapy and patients with Duchenne muscular dystrophy. Arq Neuropsiquiatr. 2012;70(3):191-5.
26 Cacchiarelli D, et al. miRNAs as serum biomarkers for Duchenne muscular dystrophy. EMBO Mol Med. 2011 May;3(5):258-65
27. http://www.mfm-nmd.org
