# Supplementary material for: Effect of Combination l-Citrulline and Metformin Treatment on Motor Function in Patients With Duchenne Muscular Dystrophy: A Randomized Clinical Trial
Source: JAMA Netw Open. 2019 Oct 30;2(10):e1914171. doi: 10.1001/jamanetworkopen.2019.14171 (PMC6824222; doi:10.1001/jamanetworkopen.2019.14171)
Supplement: Supplement 2. — eTable 1. Change in MFM D1 Subscore, Secondary End Points (Biomarkers Reported Separately), and Subgroup Analysis From Baseline to Week 26 in the Complete-Case Population eTable 2. Overall Study Results Week 13 in the ITT Population eTable 3. Changes in Biomarkers Week 26 in the ITT Population eTable 4. Changes in Biomarkers Week 13 in the ITT Population eFigure. Changes in Mean Motor Function Measure Subscore D1 at Weeks 13 and 26 Compared to Baseline [file jamanetwopen-2-e1914171-s002.pdf]

Supplementary Online Content

Hafner P, Bonati U, Klein A, et al. Effect of combination L-citrulline and metformin treatment on motor function in patients with Duchenne muscular dystrophy: a randomized clinical trial. *JAMA Netw Open*. 2019;2(10):e1914171.
doi:10.1001/jamanetworkopen.2019.14171

eTable 1. Change in MFM D1 Subscore, Secondary End Points (Biomarkers Reported Separately), and Subgroup Analysis From Baseline to Week 26 in the Complete-Case Population

eTable 2. Overall Study Results Week 13 in the ITT Population

eTable 3. Changes in Biomarkers Week 26 in the ITT Population

eTable 4. Changes in Biomarkers Week 13 in the ITT Population

eFigure. Changes in Mean Motor Function Measure Subscore D1 at Weeks 13 and 26 Compared to Baseline

This supplementary material has been provided by the authors to give readers additional information about their work.

© 2019 Hafner P et al. *JAMA Network Open*.

© 2019 Hafner P et al. *JAMA Network Open*.

eTable 1. Change in MFM D1 Subscore, Secondary End Points (Biomarkers Reported Separately), and Subgroup Analysis From Baseline to Week 26 in the Complete-Case Population

	**Cit/Met group (n=21)**			**Placebo group (n=22)**			**Group difference**	
	**Mean at baseline (SD)**	**Mean at week 26 (SD)**	**Change from baseline to week 13 (95% CI)**	**Mean at baseline (SD)**	**Meat at week 26 (SD)**	**Change from baseline to week 13 (95% CI)**	**Difference (95% CI)**	***p* value**
**MFM D1 sub-score (%)**	61.8 (11.0)	57.5 (14.0)	-4.1 (-8.8 to 0.6)	59.0 (10.8)	49.5 (14.1)	-9.6 (-14.2 to -5.1)	5.5 (-1.0 to 12.1)	0.10
**MFM total score (%)**	80.6 (6.5)	80.1 (6.2)	-0.2 (-2.6 to 2.3)	78.9 (5.6)	76.0 (7.2)	-3.2 (-5.6 to -0.8)	3.0 (-0.4 to 6.5)	0.08
**MFM D2 sub-score (%)**	96.2 (4.8)	97.0 (2.3)	1.1 (-0.3 to 2.5)	95.5 (4.2)	95.6 (3.7)	-0.2 (-1.5 to 1.2)	1.3 (-0.6 to 3.2)	0.18
**MFM D3 sub-score (%)**	90.9 (6.9)	93.0 (4.9)	2.4 (0.0 to 4.7)	89.2 (8.7)	91.3 (8.9)	1.9 (-0.4 to 4.1)	0.5 (-2.7 to 3.8)	0.76
**Strength (N)**	174.6 (66.6)	171.3 (72.7)	-3.2 (-17.3 to 10.9)	160.6 (52.1)	148.9 (61.5)	-11.8 (-25.5 to 2.0)	8.6 (-11.2 to 28.4)	0.39
**6MWD (m)**	373.0 (91.6)	339.0 (102.3)	-32.5 (-65.8 to 0.8)	358.5 (55.2)	330.8 (89.3)	-31.6 (-64.6 to 1.3)	-0.8 (-47.7 to 46.0)	0.97
**10m Walk Test (s)**	6.3 (1.8)	6.9 (2.2)	0.6 (-0.2 to 1.5)	6.6 (1.5)	7.9 (3.3)	1.4 (0.5 to 2.3)	-0.8 (-2.1 to 0.5)	0.21
**Supine up (s)**	9.0 (5.5)	11.7 (8.5)	2.5 (-1.4 to 6.5)	10.1 (6.4)	12.7 (10.6)	3.7 (-0.4 to 7.8)	-1.1 (-6.8 to 4.5)	0.69
Pre-specified subgroup analysis for the primary outcome (MFM D1 sub-score)								
**6MWD at baseline ≥350m (n=28)**	67.1 (9.2)	63.7 (9.8)	-2.9 (-7.1 to 1.4)	61.5 (12.1)	52.3 (12.7)	-9.7 (-13.6 to -5.8)	6.8 (1.0 to 12.6)	0.02
**6MWD at baseline <350m (n=15)**	53.2 (8.2)	47.4 (14.4)	-5.8 (-18.1 to 6.4)	53.5 (4.0)	43.6 (16.1)	-9.8 (-22.9 to 3.2)	4.0 (-13.9 to 21.8)	0.63
								p for interaction 0.65
Post-hoc subgroup analysis for the primary outcome (MFM D1 sub-score)								
**Usage of corticosteroids (n=31)**	59.9 (10.1)	53.4 (13.2)	-6.5 (-13.1 to 0.1)	59.6 (11.1)	50.9 (14.3)	-8.8 (-13.8 to -3.8)	2.3 (-6.0 to 10.6)	0.58
**No usage of corticosteroids (n=13)**	63.8 (12.1)	62.1 (14.0)	-1.3 (-9.1 to 6.5)	54.7 (9.7)	41.0 (11.2)	-15.3 (-30.0 to -0.6)	14.0 (-3.0 to 31.0)	0.09
								p for interaction 0.18

Abbreviations: Cit/Met= L-citrulline and metformin; SD= standard deviation; CI= confidence interval; MFM= motor function measure; D1= first dimension of the MFM, referring to the “transfer and standing posture”; D2= second dimension referring to the proximal and axial motor functions; D3= third dimension referring to the distal motor function; 6MWD= 6 minutes walking distance; QMT= quantitative muscle testing; MRI= magnetic resonance imaging; FF= fat fraction; T2= transverse relaxation times; m= meters; n= sample size

© 2019 Hafner P et al. *JAMA Network Open*.

eTable 2. Overall Study Results Week 13 in the ITT Population

	**Cit/Met group (n=23)**			**Placebo group (n=24)**			**Group difference**	
	**Mean at baseline (SD)**	**Mean at week 13 (SD)**	**Change from baseline to week 13 (95% CI)**	**Mean at baseline (SD)**	**Meat at week 13 (SD)**	**Change from baseline to week 13 (95% CI)**	**Difference (95% CI)**	***p* value**
**MFM D1 sub-score (%)**	60.1 (11.9)	59.2 (15.2)	-1.8 (-5.0 to 1.5)	58.0 (10.8)	54.6 (11.9)	-3.8 (-7.0 to -0.6)	2.1 (-2.5 to 6.6)	0.37
**MFM total score (%)**	79.4 (7.4)	80.9 (7.4)	1.1 (-1.0 to 3.2)	78.7 (5.5)	77.7 (6.6)	-1.1 (-3.1 to 0.9)	2.2 (-0.7 to 5.1)	0.14
**MFM D2 sub-score (%)**	95.7 (5.3)	96.2 (5.0)	0.4 (-1.5 to 2.3)	95.6 (4.0)	96.0 (4.6)	0.4 (-1.3 to 2.2)	-0.1 (-2.6 to 2.5)	0.96
**MFM D3 sub-score (%)**	89.9 (9.0)	94.2 (6.0)	3.8 (1.7 to 5.8)	89.7 (8.6)	90.3 (7.1)	0.8 (-1.1 to 2.8)	2.9 (0.1 to 5.8)	0.04
**Strength (N)**	164.8 (71.6)	177.7 (73.8)	8.8 (-2.0 to 19.7)	156.4 (51.8)	149.0 (51.4)	-9.4 (-20.1 to 1.3)	18.3 (3.0 to 33.5)	0.02
**6MWD (m)**	362.0 (94.7)	362.6 (91.6)	-4.0 (-22.5 to 14.5)	356.7 (55.3)	336.5 (74.0)	-19.2 (-37.3 to -1.2)	15.2 (-10.7 to 41.2)	0.24
**10m Walk Test (s)**	6.7 (2.2)	6.8 (1.9)	0.2 (-0.2 to 0.6)	6.8 (1.5)	7.1 (2.0)	0.4 (0.0 to 0.8)	-0.2 (-0.7 to 0.4)	0.48
**Supine up (s)**	10.1 (6.6)	10.7 (8.1)	1.00 (-2.0 to 4.0)	10.4 (6.2)	12.4 (10.0)	2.7 (-0.3 to 5.6)	-1.7 (-5.8 to 2.5)	0.43
Pre-specified subgroup analysis for the primary outcome (MFM D1 sub-score)								
**6MWD at baseline ≥350m (n=29)**	67.1 (9.2)	66.9 (12.9)	-0.0 (-4.0 to 4.0)	60.7 (12.1)	58.3 (11.9)	-3.2 (-6.9 to 0.5)	3.1 (-2.4 to 8.7)	0.25
**6MWD at baseline <350m (n=18)**	51.0 (8.6)	48.1 (11.2)	-3.6 (-10.1 to 2.8)	52.6 (4.5)	47.7 (8.9)	-4.6 (-11.5 to 2.3)	0.9 (-8.5 to 10.4)	0.83
								p for interaction 0.58
Post-hoc subgroup analysis for the primary outcome (MFM D1 sub-score)								
**Usage of corticosteroids (n=33)**	58.5 (10.8)	54.5 (13.1)	-4.1 (-8.0 to -0.1)	58.5 (11.1)	55.6 (12.1)	-3.4 (-6.5 to -0.3)	-0.7 (-5.7 to 4.4)	0.78
**No usage of corticosteroids (n=14)**	61.8 (13.4)	64.9 (16.3)	2.1 (-5.4 to 9.7)	54.70 (9.7)	47.9 (9.0)	-7.3 (-20.8 to 6.2)	9.5 (-6.4 to 25.4)	0.21
								p for interaction 0.15

Abbreviations: Cit/Met= L-citrulline and metformin; SD= standard deviation; CI= confidence interval; MFM= motor function measure; D1= first dimension of the MFM, referring to the “transfer and standing posture”; D2= second dimension referring to the proximal and axial motor functions; D3= third dimension referring to the distal motor function; 6MWD= 6 minutes walking distance; QMT= quantitative muscle testing; MRI= magnetic resonance imaging; FF= fat fraction; T2= transverse relaxation times; m= meters; n= sample size

© 2019 Hafner P et al. *JAMA Network Open*.

eTable 3. Changes in Biomarkers Week 26 in the ITT Population

	Cit/Met group (n=23)			Placebo group (n=24)			Group difference	
	**Mean at baseline (SD)**	**Mean at week 26 (SD)**	**Change from baseline (95% CI)**	**Mean at baseline (SD)**	**Mean at week 26 (SD)**	**Change from baseline(95% CI)**	**Difference (95% CI)**	***p* value**
**S CK (U/l)**	13574.1 (6946.3)	10399.4 (5363.0)	-2334.1 (-4867.7 to 199.6)	12443.4 (8976.2)	12640.0 (6876.6)	-356.5 (-2978.7 to 2265.6)	-1977.5 (-5622.6 to 1667.6)	0.28
**S CK MB (µg/l)**	332.9 (187.2)	265.6 (128.4)	-49.8 (-101.6 to 2.1)	304.5 (192.4)	310.5 (125.5)	-7.2 (-57.4 to 42.9)	-42.5 (-114.5 to 29.5)	0.24
**S ASAT (U/l)**	280.7 (140.4)	214.7 (98.5)	-64.3 (-105.5 to -23.2)	289.5 (147.1)	271.8 (102.7)	-15.8 (-57.0 to 25.3)	-48.5 (-106.8 to 9.8)	0.10
**S ALAT (U/l)**	377.7 (165.1)	311.1 (156.8)	-56.0 (-105.2 to -6.9)	402.8 (136.0)	382.7 (146.2)	-16.5 (-65.1 to 32.2)	-39.6 (-109.1 to 29.9)	0.26
**S LDH (U/l)**	1170.7 (423.2)	978.5 (404.3)	-145.0 (-272.6 to -17.5)	1090.3 (409.2)	1074.5 (312.2)	-38.1 (-164.8 to 88.6)	-106.9 (-286.1 to 72.2) -1.0	0.23
**S GGT (U/l)**	10.6 (2.7)	10.1 (3.2)	-0.5 (-1.6 to 0.6)	9.9 (2.7)	10.3 (2.7)	0.5 (-0.7 to 1.7)	(-2.6 to 0.6)	0.21
**S Bilirubin (µmol/l)**	7.5 (6.5)	6.0 (5.3)	-1.2 (-2.2 to -0.2)	4.8 (2.7)	4.2 (2.5)	-1.2 (-2.2 to -0.2)	0.0 (-1.4 to 1.5)	0.98
**S alkaline Phosphatase (U/l)**	100.3 (25.6)	91.2 (25.3)	-9.1 (-16.0 to -2.3)	101.4 (35.8)	99.8 (34.7)	-0.1 (-7.2 to 6.9)	-9.0 (-18.9 to 0.8)	0.07
**S Creatinine (µmol/l)**	15.0 (3.8)	16.2 (5.8)	0.9 (-0.8 to 2.6)	16.1 (4.5)	16.2 (4.3)	-0.0 (-1.7 to 1.6)	0.9 (-1.4 to 3.3)	0.43
**U sum BCAA (mmol/mol)**	24.8 (8.6)	23.1 (6.1)	-1.6 (-5.4 to 2.2)	24.7 (6.2)	25.0 (11.0)	0.3 (-3.4 to 4.0)	-1.9 (-7.2 to 3.4)	0.47
**U sum EAA (mmol/mol)**	331.0 (141.0)	294.7 (133.7)	-44.7 (-137.3 to 47.8)	354.3 (104.6)	385.0 (278.3)	38.9 (-51.7 to 129.5)	-83.7 (-213.4 to 46.1)	0.20
**U sum NEAA (mmol/mol)**	1105.7 (453.6)	866.6 (314.2)	-279.8 (-474.1 to -85.4)	1210.3 (369.9)	1127.2 (574.1)	-44.2 (-234.4 to 146.1)	-235.6 (-508.7 to 37.5)	0.09
**U sum all (mmol/mol)**	1471.5 (573.6)	1208.7 (447.1)	-313.8 (-595.7 to -31.9)	1598.9 (438.1)	1549.5 (827.3)	-0.5 (-276.5 to 275.4)	-313.2 (-709.3 to 82.8)	0.12
**U sum all without NAA (mmol/mol)**	1461.5 (572.8)	1184.4 (432.8)	-328.2 (-606.4 to -49.9)	1589.3 (438.1)	1537.3 (821.0)	-3.0 (-275.3 to 269.3)	-325.2 (-716.1 to 65.7)	0.10

Abbreviations: Cit/Met= L-citrulline and metformin; SD= standard deviation; CI= confidence interval; S= serum; CK= Creatinekinase; CK-MB = Creatinekinase-MB isotype; ASAT= Aspartate aminotransferase; ALAT = Alanine aminotransferase; LDH= lactate dehydrogenase; GGT= gamma-glutamyltransferase; U = Urine; sum = summated; BCAA = branched chain amino acids; EAA= essential amino acids; NEAA= non-essential amino acids, NAA = nitroergic amino acids (Ornithin, Citrulline, Arginine)

© 2019 Hafner P et al. *JAMA Network Open*.

eTable 4. Changes in Biomarkers Week 13 in the ITT Population

	Cit/Met group (n=23)			Placebo group (n=24)			Group difference	
	**Mean at baseline (SD)**	**Mean at week 13 (SD)**	**Change from baseline (95% CI)**	**Mean at baseline (SD)**	**Mean at week 13 (SD)**	**Change from baseline (95% CI)**	**Difference (95% CI)**	***p* value**
**S CK (U/l)**	13574.1 (6946.3)	11120.7 (5548.2)	-1938.5 (-4446.5 to 569.5)	12443.4 (8976.2)	12351.4 (7333.8)	-524.8 (-3058.7 to 2009.1)	-1413.7 (-4974.7 to 2147.3)	0.43
**S CK MB (µg/l)**	332.9 (187.2)	284.6 (146.6)	-34.6 (-81.1 to 11.8)	304.5 (192.4)	284.2 (107.2)	-27.6 (-73.2 to 18.0)	-7.1 (-72.3 to 58.1)	0.83
**S ASAT (U/l)**	280.7 (140.4)	233.4 (99.5)	-50.7 (-92.6 to -8.7)	289.5 (147.1)	272.8 (118.1)	-12.8 (-55.3 to 29.8)	-37.9 (-97.8 to 22.0)	0.21
**S ALAT (U/l)**	377.7 (165.1)	335.8 (147.2)	-44.8 (-85.8 to -3.7)	402.8 (136.0)	397.6 (147.8)	-2.8 (-43.8 to 38.2)	-42.0 (-100.1 to 16.2)	0.15
**S LDH (U/l)**	1170.7 (423.2)	1059.2 (393.3)	-99.7 (-209.7 to 10.2)	1090.3 (409.2)	1054.5 (349.7)	-43.7 (-149.6 to 62.2)	-56.0 (-209.2 to 97.1)	0.46
**S GGT (U/l)**	10.6 (2.7)	10.1 (2.4)	-0.5 (-1.4 to 0.4)	9.9 (2.7)	11.0 (3.4)	1.1 (0.1 to 2.1)	-1.6 (-3.0 to -0.3)	0.02
**S Bilirubin (µmol/l)**	7.5 (6.5)	6.8 (5.8)	-0.7 (-1.7 to 0.2)	4.8 (2.7)	4.5 (2.9)	-0.6 (-1.5 to 0.4)	-0.2 (-1.5 to 1.2)	0.81
**S alkaline Phosphatase (U/l)**	100.3 (25.6)	94.0 (19.2)	-7.3 (-14.0 to -0.7)	101.4 (35.8)	96.9 (26.9)	-1.6 (-8.2 to 5.0)	-5.7 (-15.1 to 3.6)	0.22
**S Creatinine (µmol/l)**	15.0 (3.8)	15.9 (4.3)	0.6 (-0.8 to 2.0)	16.1 (4.5)	16.7 (3.5)	0.7 (-0.7 to 2.1)	-0.1 (-2.1 to 1.8)	0.89
**U sum BCAA (mmol/mol)**	24.8 (8.6)	22.7 (9.1)	-2.1 (-6.2 to 1.9)	24.7 (6.2)	23.8 (11.0)	-0.9 (-4.9 to 3.1)	-1.2 (-6.9 to 4.5)	0.67
**U sum EAA (mmol/mol)**	331.0 (141.0)	279.3 (150.2)	-57.7 (-117.9 to 2.5)	354.3 (104.6)	335.6 (156.6)	-12.9 (-71.8 to 46.1)	-44.8 (-129.3 to 39.6)	0.29
**U sum NEAA (mmol/mol)**	1105.7 (453.6)	794.6 (391.4)	-343.1 (-515.5 to -170.7)	1210.3 (369.9)	1048.5 (475.0)	-131.1 (-299.9 to 37.6)	-212.0 (-454.2 to 30.3)	0.08
**U sum all (mmol/mol)**	1471.5 (573.6)	1122.3 (546.5)	-386.6 (-618.7 to -154.5)	1598.9 (438.1)	1418.2 (619.0)	-144.8 (-372.0 to 82.4)	-241.8 (-567.9 to 84.3)	0.14
**U sum all without NAA (mmol/mol)**	1461.5 (572.8)	1096.6 (538.8)	-402.3 (-632.0 to -172.7)	1589.3 (438.1)	1408.0 (616.0)	-145.5 (-370.3 to 79.3)	-256.8 (-579.5 to 65.9)	0.12

Abbreviations: Cit/Met= L-citrulline and metformin; SD= standard deviation; CI= confidence interval; S= serum; CK= Creatinekinase; CK-MB = Creatinekinase-MB isotype; ASAT= Aspartate aminotransferase; ALAT = Alanine aminotransferase; LDH= lactate dehydrogenase; GGT= gamma-glutamyltransferase; U = Urine; sum = summated; BCAA = branched chain amino acids; EAA= essential amino acids; NEAA= non-essential amino acids, NAA = nitroergic amino acids (Ornithin, Citrulline, Arginine)

© 2019 Hafner P et al. *JAMA Network Open*.

eFigure. Changes in Mean Motor Function Measure Subscore D1 at Weeks 13 and 26 Compared to Baseline

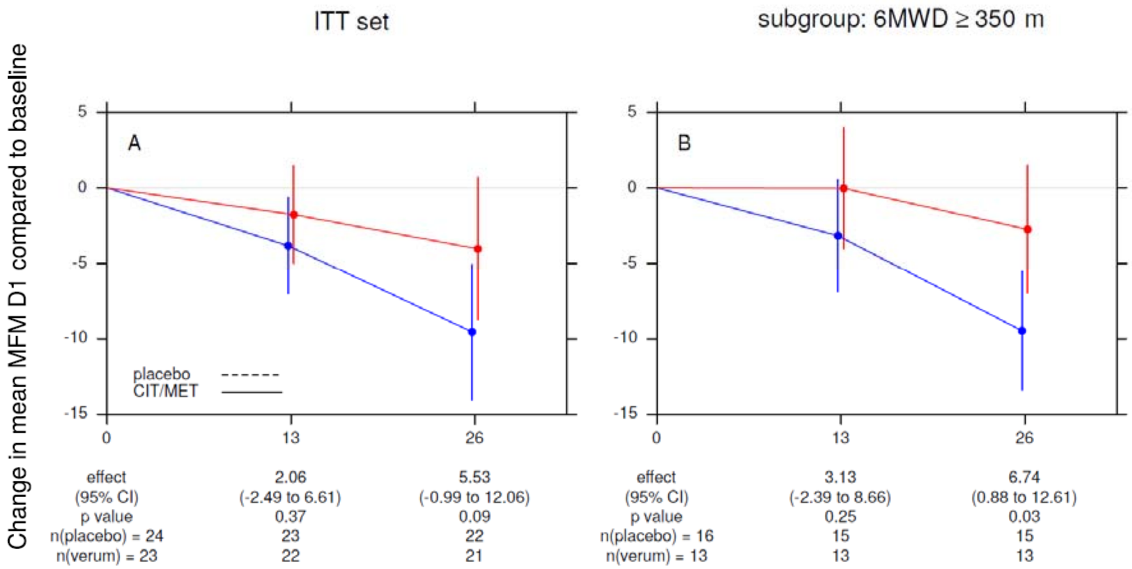

(A) in the intention-to-treat population and (B) for the stable sub-group with a 6 minutes walking distance ≥ 350 m at baseline.

© 2019 Hafner P et al. *JAMA Network Open*.
