# Supplementary material for: Effect of Combination l-Citrulline and Metformin Treatment on Motor Function in Patients With Duchenne Muscular Dystrophy: A Randomized Clinical Trial
Source: JAMA Netw Open. 2019 Oct 30;2(10):e1914171. doi: 10.1001/jamanetworkopen.2019.14171 (PMC6824222; doi:10.1001/jamanetworkopen.2019.14171)
Supplement: Supplement 3. — Data Sharing Statement [file jamanetwopen-2-e1914171-s003.pdf]

# Data Sharing Statement

Hafner P, Bonati U, Klein A, et al. Effect of combination L-citrulline and metformin treatment on motor function in patients with Duchenne muscular dystrophy: a randomized clinical trial. *JAMA Netw Open*. 2019;2(10):e1914171. doi:10.1001/jamanetworkopen.2019.14171

## Data

**Data available:** No
